# Spontaneous and frequent conformational dynamics induced by A…A mismatch in d(CAA)·d(TAG) duplex

**DOI:** 10.1038/s41598-021-82669-4

**Published:** 2021-02-11

**Authors:** Yogeeshwar Ajjugal, Kripi Tomar, D. Krishna Rao, Thenmalarchelvi Rathinavelan

**Affiliations:** 1 Department of Biotechnology, Indian Institute of Technology Hyderabad, Kandi, Sangareddy District, Telangana State 502285 India; 2 Tata Institute of Fundamental Research, 36/P, Gopanpally Mandal, Ranga Reddy District, Hyderabad, Telangana State 500107 India

**Keywords:** Biophysics, Structural biology

## Abstract

Base pair mismatches in DNA can erroneously be incorporated during replication, recombination, etc. Here, the influence of A…A mismatch in the context of 5′CAA·5′TAG sequence is explored using molecular dynamics (MD) simulation, umbrella sampling MD, circular dichroism (CD), microscale thermophoresis (MST) and NMR techniques. MD simulations reveal that the A…A mismatch experiences several transient events such as base flipping, base extrusion, etc*.* facilitating B–Z junction formation. A…A mismatch may assume such conformational transitions to circumvent the effect of nonisostericity with the flanking canonical base pairs so as to get accommodated in the DNA. CD and 1D proton NMR experiments further reveal that the extent of B–Z junction increases when the number of A…A mismatch in d(CAA)·d(T(A/T)G) increases (1–5). CD titration studies of d(CAA)·d(TAG)_n=5_ with the hZα_ADAR1_ show the passive binding between the two, wherein, the binding of protein commences with B–Z junction recognition. Umbrella sampling simulation indicates that the mismatch samples *anti…*+ *syn/*+ *syn…anti, anti…anti & *+ *syn…*+ *syn glycosyl* conformations. The concomitant spontaneous transitions are: a variety of hydrogen bonding patterns, stacking and minor or major groove extrahelical movements (with and without the engagement of hydrogen bonds) involving the mismatch adenines*.* These transitions frequently happen in *anti…anti* conformational region compared with the other three regions as revealed from the lifetime of these states. Further, 2D-NOESY experiments indicate that the number of cross-peaks diminishes with the increasing number of A…A mismatches implicating its dynamic nature. The spontaneous extrahelical movement seen in A…A mismatch may be a key pre-trapping event in the mismatch repair due to the accessibility of the base(s) to the sophisticated mismatch repair machinery.

## Introduction

Mismatch emerges in the DNA when two non-complementary bases align together as a base pair (known as non-canonical or non-Watson–Crick base pair)^1^ either endogenously during the biological processes like DNA replication^2^, recombination, spontaneous deamination, etc.^3–5^ or exogenously by ionizing radiations such as X-rays and gamma rays^2,6^, and by the action of certain chemical compounds known as mutagenic agents. The mismatches often pose a major challenge to maintain genome integrity and can lead to deleterious conditions like neurological disorders^7^ and cancer^8–10^.

DNA mismatches are the root cause for the trinucleotide repeat expansion disorders (TREDs), wherein, the trinucleotide microsatellite forms unusual secondary structures consisting of periodic base pair mismatches^11^. For instance, overexpansion of CAG trinucleotide repeats in the human forms hairpin structure with periodic A…A mismatches^12,13^ and leads to neurological disorders like Huntington’s disease and several spinocerebellar ataxias^13–16^. Nonetheless, unlike the other seven non-canonical base pairs, atomistic details about the influence of an A…A mismatch on the DNA conformation is not well understood. Until now, the structural information of the A…A mismatch in a DNA duplex is available only in the context of its complex with the mismatch repair protein (PDB ID: 2WTU, 1OH6), DNA polymerase beta (PDB ID: 5J0O, 1ZJM and 5J29), rhodium ((PDB ID: 3GSJ), napthyridine-azaquinolone (PDB ID: 1X26) and delta-[Ru(bpy)2dppz]2 + (PDB ID:4E1U). The earlier NMR investigations have revealed that the A…A mismatch induces local structural distortions in the DNA duplex^17,18^. The CD and MD investigations of the DNA duplexes comprising A…A mismatch, wherein, the A…A mismatch is embedded in the CAG and GAC sequences, indicate that the A…A mismatch induces B–Z junction^19,20^.

Here, the influence of the A…A mismatch that is sandwiched between a 5′C…G and 3′A…T base pairs in a 5′CAA·5′TAG sequence has been investigated. The CAA·TTG microsatellites are also found in the exonic regions of the human genome, although they are underrepresented compared with CNG (N = A, T, G, C) repeats^21^. CAA·TTG tandem repeat markers are also present significantly in the plant genome^22,23^. It is noteworthy that the secondary structure of such tandem repeats in the intergenic regions can alter the chromatin and influence the expression of the nearby genes^24,25^. Interestingly, a recent study has shown that the CAA·TTG repeats are present along with the CAG repeats in the zinc finger homeobox 3 (*ZFHX3*) gene that is associated with coronary heart disease in Chinese population^26^. It is also well known that CAA interrupts in CAG repeats decreases the repeat expansion^27^. An earlier NMR and MD studies on the DNA duplex occurring at codon 12 (a mutational hotspot) of the *KRAS* gene indicates that the A…A mismatch flanked by 5′C…G and 3′A…T base pairs is engaged in an N6…N1 hydrogen bond^18^. However, the detailed information on the influence of the A…A mismatch on the conformation of the DNA is not well understood in the context of the CAA sequence. Thus, it is important to investigate the influence of the A…A mismatch in the context of the CAA·TTG DNA sequence.

The molecular dynamics simulation, umbrella sampling MD, circular dichroism, microscale thermophoresis and NMR experiments carried out here to investigate the conformational preference of the A…A mismatch in the CAA sequence indicate that A…A mismatch is highly dynamic in nature. Spontaneous and frequent transitions between base flipping, extrusion, stacking and a variety of hydrogen bond conformations are observed concomitant with the formation of B–Z junction during the MD and umbrella sampling simulations. Such a dynamic nature of A…A mismatch is confirmed by the 2D-NOESY experiment, wherein, the number of proton-proton cross-peaks decreases with the increasing number of A…A mismatch. Further, CD and 1D proton NMR experiments revealed that the B–Z junction is pronounced in the duplex when the number of A…A mismatch increases. The B–Z junction formation further facilitates the binding with Z-DNA binding domain of human-ADAR1 (hZα_ADAR1_) protein as seen in CD and microscale thermophoresis experiments. Such an aberrant backbone conformational preference along with the extrahelical minor or major groove movement of the adenine(s) may be the key structural features responsible for the recognition of A…A mismatch by the repair proteins to initiate the chemical reaction.

## Results

As the primary aim of this investigation is to explore the influence of the A…A mismatch in the midst of d(CAA) sequence, 500 ns MD simulation has been carried out for d(CAA)_5_·d(T(A/T)G)_5_, wherein, the central 5′CAA·5′TAG encompasses a single A…A mismatch (Table 1, Scheme DCA-1). Two starting *glycosyl* conformations have been considered for the mismatch: A_8_(*anti*)…A_23_(*anti*) and A_8_(*anti*)… A_23_(+ *syn*)/A_8_(+ *syn*)… A_23_(*anti*)^19,20, 28–31^. A_8_…A_23_ mismatch is modeled to be involved in N1…N6 hydrogen bond.

**Table 1:** DNA duplexes used in the current investigation.

Number of A…A mismatches	Scheme	Sequences
1	DCA-1	
2	DCA-2	
3	DCA-3	
4	DCA-4	
5	DCA-5	
0	WC	
1	DCA-1a	
1	DAC-1a	

The canonical and the non-canonical base pairs are indicated by “|” and “*” respectively.

### A…A mismatch in the midst of CAA sequence induces B–Z junction

**Figure 1:**
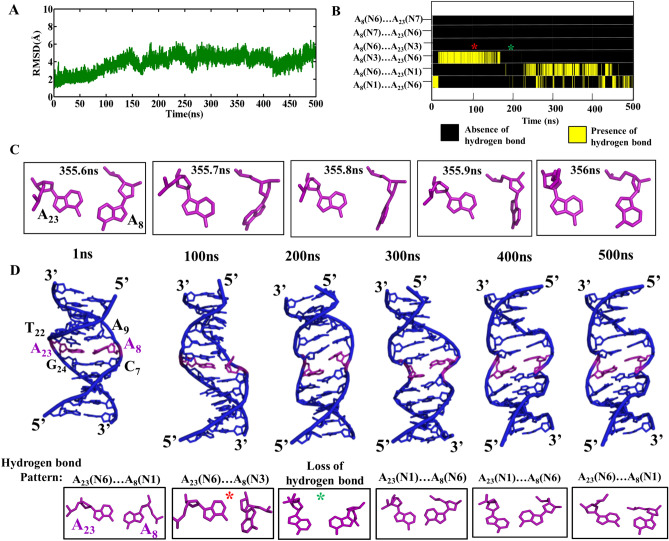
A_8_…A_23_ mismatch with *anti…anti* starting *glycosyl* conformation induces B–Z junction in the DNA duplex (Scheme DCA-1, Table 1). (**A**) Time vs RMSD profile. The MATLAB 7.11.0 software (www.mathworks.com) was used to plot the data. (**B**) A_8_…A_23_ hydrogen bond lifetime profile. The possible hydrogen bonding patterns between A_8_ and A_23_ are indicated in the Y-axis. Note that the red and green colored stars are associated with the transient events represented in (**D**). The GNUPLOT 5.2 software was used to plot the data^54^. (**C**) Transient flipping of A_8_ observed around 356 ns. (**D**) Cartoon representation of the conformational dynamics (top) and the concomitant A_8_…A_23_ hydrogen bond dynamics (boxed, bottom). The figures (**C**) and (**D**) were generated by using Pymol 1.3 (www.pymol.com) software^53^.

**Figure 2:**
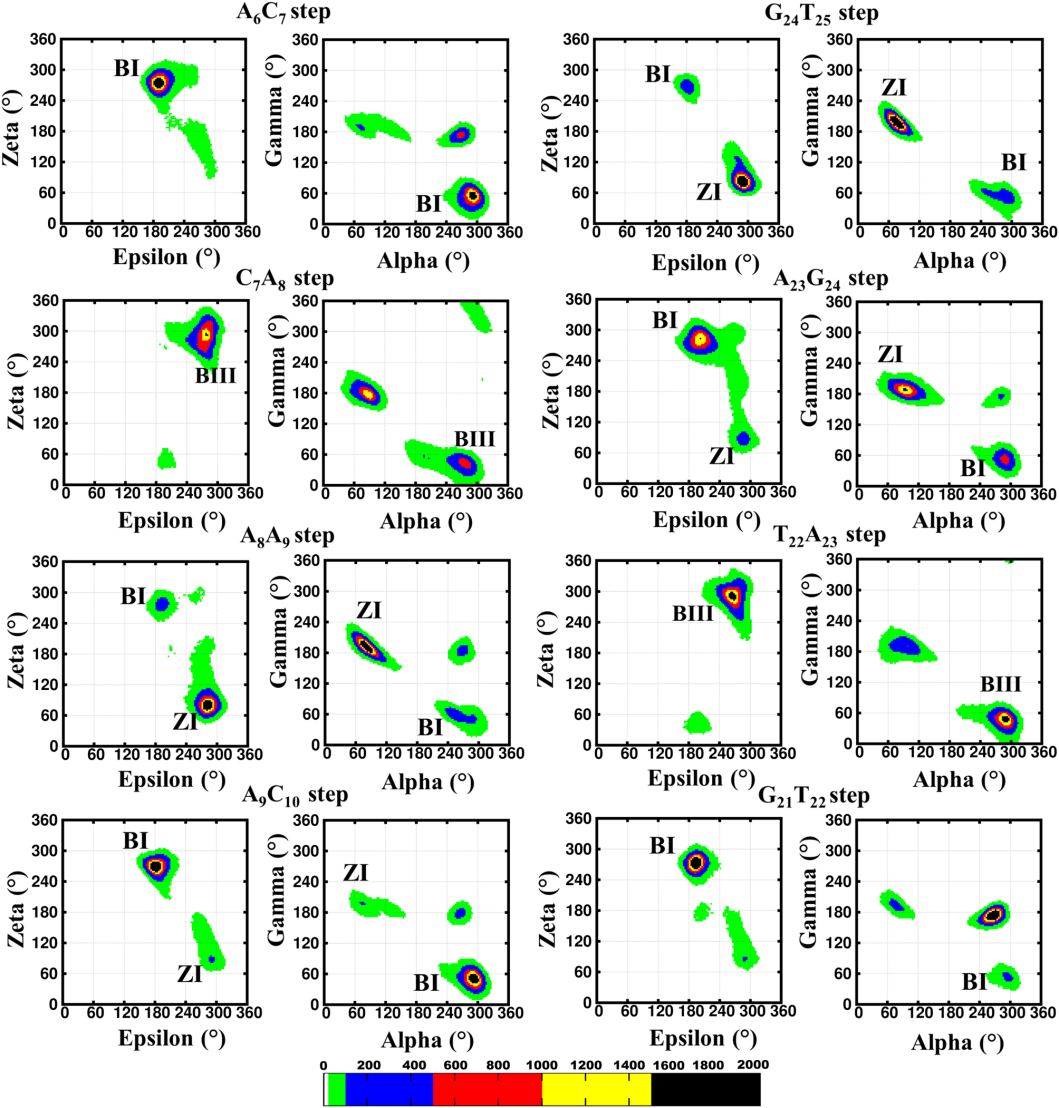
The backbone torsion angles (ε, ζ, α, γ) corresponding to the central pentamer that encompass A_8_*(anti)…A*_*23*_*(anti)* mismatch (scheme DCA-1). (ε&ζ) (1st and 3rd column) and (α&γ) (2nd and 4th column) 2D contour density plots corresponding to various steps in the vicinity of the mismatch. Note that the BI ((ε, ζ, α, γ) = (t, g^−^, g^−^, g^+^)), BII (g^−^, t, g^−^, g^+^), BIII (g^−^, g^−^, g^−^, g^+^) and ZI (g^−^, g^+^, g^+^, t) conformations are indicated adjacent to the corresponding regions. Other conformational intermediates can also be seen in the plot. The trajectories corresponding to the last 300 ns simulation is considered for the plotting. The scale corresponding to the isolines is given at the bottom. The GNUPLOT 5.2 software was used to plot the data^54^.

The root mean square deviation (RMSD) calculated for the DCA-1, wherein, the A…A mismatch is modeled to have *anti*(A_8_)…*anti(*A_23_) *glycosyl* conformation, exhibits an average value of 4.3 Å with respect to the initial structure over the last 350 ns simulation (Fig. 1A). Beyond 12 ns, A_8_(N1)…A_23_(N6) hydrogen bond is lost and both the A’s are engaged in N3…N6 hydrogen bond. This is facilitated by the movement of one of the A’s towards the minor groove and the other A towards the major groove, which is retained until 150 ns (Fig. 1B,D with a red star). In addition to this, a total loss of hydrogen bond is observed during 150–200 ns (Fig. 1B,D with a green star). Beyond 250 ns, the hydrogen bond dynamics is between N1(A_8_)…N6(A_23_) and N6(A_8_)…N1(A_23_) which can be seen in the hydrogen bond lifetime analysis (Fig. 1B). A total loss of hydrogen bond is also seen occasionally during the last 250 ns. Interestingly, the base (A_8_) flipping event is also seen ~ 350 ns preceded by the base-pair opening (Fig. 1C, Movie S1). Such a base pair dynamics significantly distorts the backbone geometry around the mismatch site, leading to the widened or narrowed minor groove (Fig. 1D). This subsequently results in a slightly higher RMSD after 150 ns (Fig. 1A). The backbone conformational angles (ε, ζ, α, γ) at C_7_A_8_, A_8_A_9_, T_22_A_23_, A_23_G_24_ and G_24_T_25_ base steps favor a variety of conformations apart from the canonical BI(t, g^−^, g^−^, g^+^) and BII(g^−^, t, g^−^, g^+^) conformations. The occurrence of BIII (g^−^, g^−^, g^−^, g^+^) conformation is observed at C_7_A_8_ and T_22_A_23_ base steps, whereas, a local ZI (g^−^, g^+^, g^+^, t) conformation is seen at the A_8_A_9_, A_9_C_10_, A_23_G_24_ and G_24_T_25_ steps (Fig. 2). Additionally, these steps take up BI conformation. Other than these conformations, a few other conformations are also seen (Fig. 2). Further, *glycosyl* torsion angles corresponding to A_8_ and A_23_ favor −*syn* conformation instead of the starting *anti* conformation after 150 ns (Supplementary Fig. S1A,B). Together, these results indicate the formation of a local B–Z junction at the A_8_…A_23_ site.

**Figure 3:**
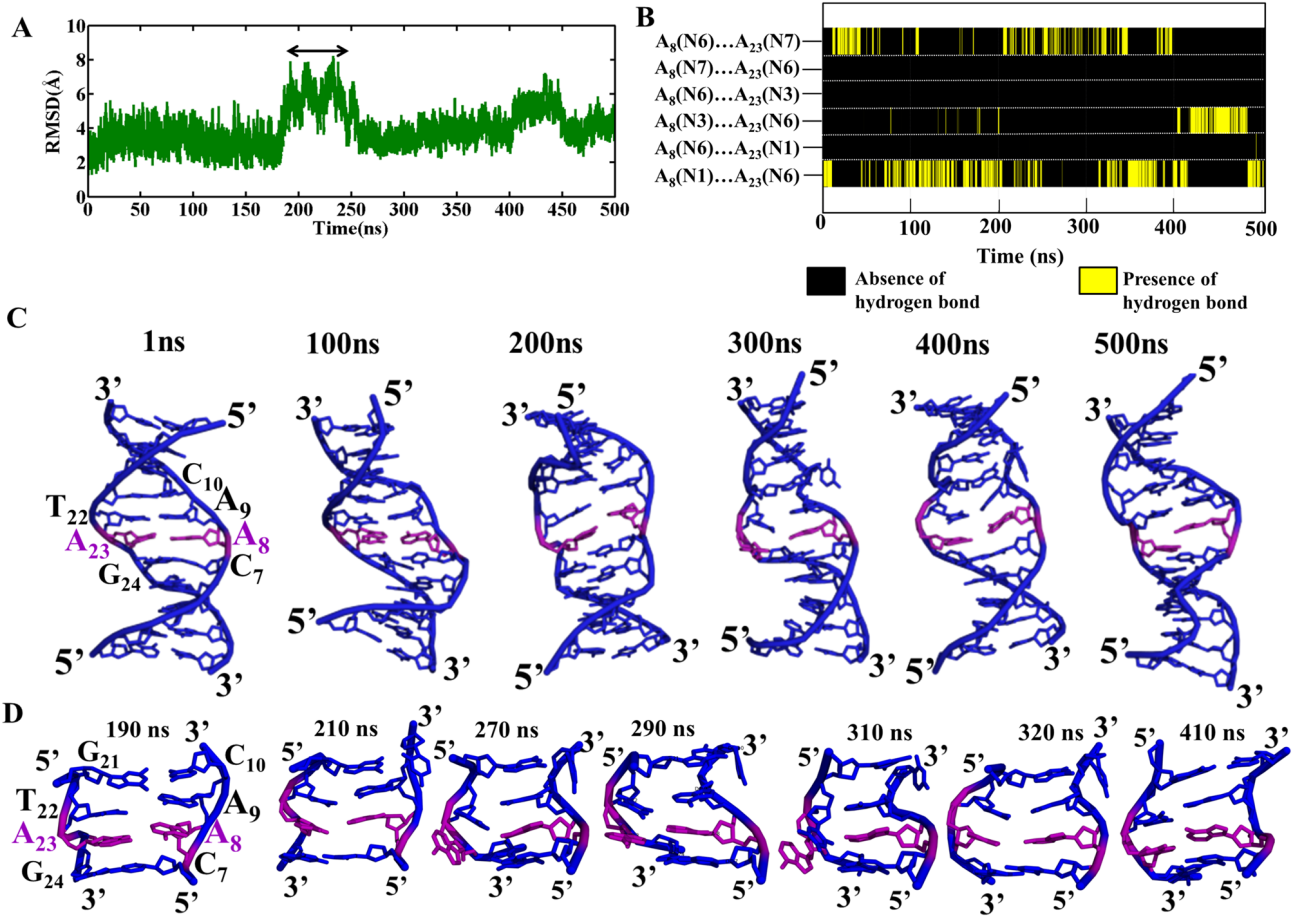
Analysis of DCA-1 DNA duplex that has *anti*… + *syn* starting *glycosyl* conformation for A_8_…A_23_. (**A**) Time vs RMSD profile. The double headed arrow indicates the increase in the RMSD during 190–250 ns. The MATLAB 7.11.0 software (www.mathworks.com) was used to plot the data. (**B**) Life time (X-axis) of different A_8_…A_23_ hydrogen bond schemes (Y-axis). The GNUPLOT 5.2 software was used to plot the data^54^. (**C**) Snapshots showing the local B–Z junction formation at the mismatch site. (**D**) The local conformational dynamics associated with the increase in RMSD (**A**). Note: A_8_…A_23_ mismatch is represented in purple color in the cartoon representation. The figures (**C**) and (**D**) were generated by using Pymol 1.3 (www.pymol.com) software^53^.

**Figure 4:**
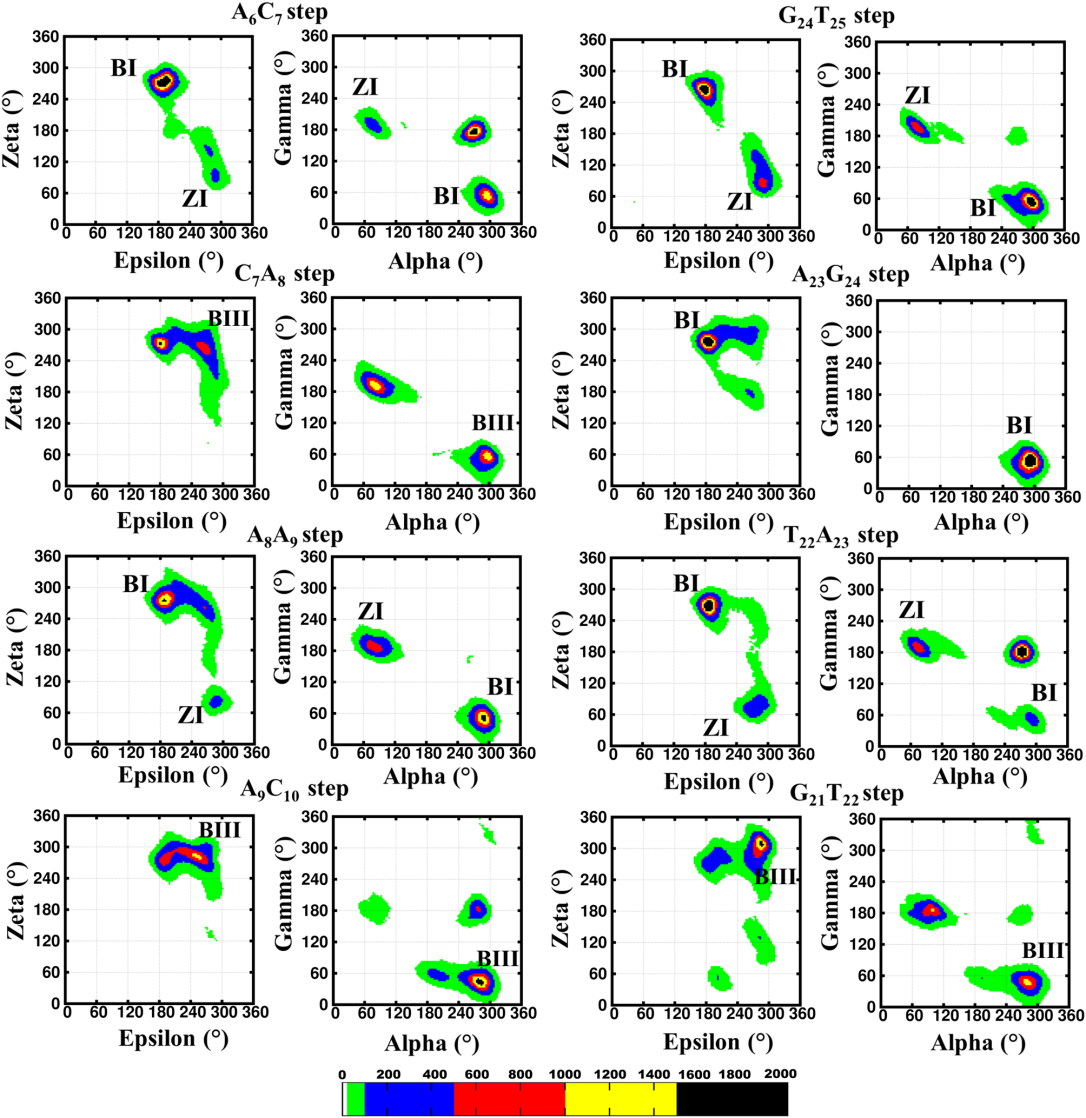
The backbone torsion angles (ε, ζ, α, γ) corresponding to the central pentamer that encompass A_8_*(anti)…A*_*23*_*(*+ *syn)* mismatch (scheme DCA-1). (ε&ζ) (1st and 3rd column) and (α&γ) (2nd and 4th column) 2D contour density plots corresponding to various steps in the vicinity of the mismatch. Note that the BI ((ε, ζ, α, γ) = (t, g^−^, g^−^, g^+^)), BII (g^−^, t, g^−^, g^+^), BIII (g^−^, g^−^, g^−^, g^+^) and ZI (g^−^, g^+^, g^+^, t) conformations are indicated adjacent to the corresponding regions. Other conformational intermediates can also be seen in the plot. The trajectories corresponding to the last 300 ns simulation is considered for the plotting. The scale corresponding to the isolines is given at the bottom. The GNUPLOT 5.2 software was used to plot the data^54^.

Analysis of the MD trajectories corresponding to the *anti…* + *syn glycosyl* conformation for A_8_…A_23_ mismatch reveals the local B–Z junction formation at the mismatch site (Fig. 3). This is accompanied by an average RMSD value of 4.2 Å (calculated over the last 350 ns) with respect to the starting conformation (Fig. 3A). However, there is an increase in the average RMSD to ~ 5.7 Å (0.8 Å) during 190–250 ns (Fig. 3A, double-headed arrow). Such an increase in the RMSD value can be attributed to the backbone conformational changes that occur at the mismatch site, which eventually leads to helical unwinding (Fig. 3C). Notably, the frequent exchange between N6(A_8_)…N7(A_23_) and N1(A_8_)…N6(A_23_) hydrogen bond is observed during the 500 ns simulation (Fig. 3B). Like in the previous situation, N3(A_8_)…N6(A_23_) hydrogen bond is also observed here. Further, a total loss of hydrogen bond is seen during the simulation as in the previous case. Very interestingly, the structural distortions at the mismatch site propagate to the neighborhood in such a way that the flanking C_10_ residue in the complementary strand undergoes extrusion at the major groove side at the cost of the canonical hydrogen bond. Concomitantly, A_23_ also undergoes extrusion towards the major groove. During 210–320 ns both C_10_ and A_23_ involve in such an extrusion event, beyond which, they resume the hydrogen bond with the respective bases as depicted in Fig. 3D. It is worth noting that such base extrusions at the B–Z junction have been reported in earlier investigations (PDB ID: 2ACJ^32,33^). The backbone conformational angles (ε, ζ, α, γ) at C_7_A_8_, A_8_A_9_, A_9_C_10_, G_21_T_22_, T_22_A_23_ and G_24_T_25_ base steps predominantly favor BIII and ZI conformations. Besides, these steps also take other conformations that are intermediate to Z-DNA and B-DNA (Fig. 4). The *glycosyl* torsion angles corresponding to A_8_…A_23_ predominantly fall in *anti…* + *syn* conformation (Supplementary Fig. S1C,D).

The MD simulation carried out by swapping the initial *glycosyl* conformation of the mismatch (viz*.,* A_8_(anti)….A_23_(+ syn)) to A_8_(+ syn)….A_23_(anti)) essentially reflects the same conformational rearrangements (Supplementary Figs. S2 and S3). This reflects in the RMSD, which fluctuates between 2 and 5 Å beyond ~ 125 ns (Supplementary Fig. S2A,C). The N7(A_8_)…N6(A_23_) hydrogen bond is highly favored, followed by N6(A_8_)…N1(A_23_) and N6(A_8_)…N3(A_23_). The A_8_…A_23_ *glycosyl* conformation predominantly samples + *syn…anti* region. However, a minor population of + *syn…-syn* conformation is also observed (Supplementary Fig. S2D). Not surprisingly, the backbone conformational angles (ε, ζ, α, γ) at the mismatch site favor BIII and ZI conformations as discussed above (Supplementary Figs. S2C and S3). Thus, the MD results clearly indicate the formation of a local B–Z junction at the A_8_…A_23_ site irrespective of the starting *glycosyl* conformation.

### Canonical base pairs in the 5′CAA·5′TTG sequence retain B-form geometry

A control simulation carried out (Scheme WC, contains only the canonical base pairs) to show that the aforementioned conformational rearrangements are the sole influence of A_8_…A_23_ mismatch indicates the retention of B-form geometry. The RMSD stays ~ 3 Å during the simulation (Supplementary Fig. S4A,B) and the canonical hydrogen bond (G…C and A….T) distances are retained (falling in the range of 2.5–3.5 Å (Supplementary Fig. S4C,D)). Further, the backbone conformational angles (ε, ζ, α, γ) predominantly favor the BI conformation (Supplementary Fig. S5). Thus, it is clear that the canonical duplex favor B-form geometry.

#### The extent of B–Z junction increases with the increasing number of A…A mismatch

**Figure 5:**
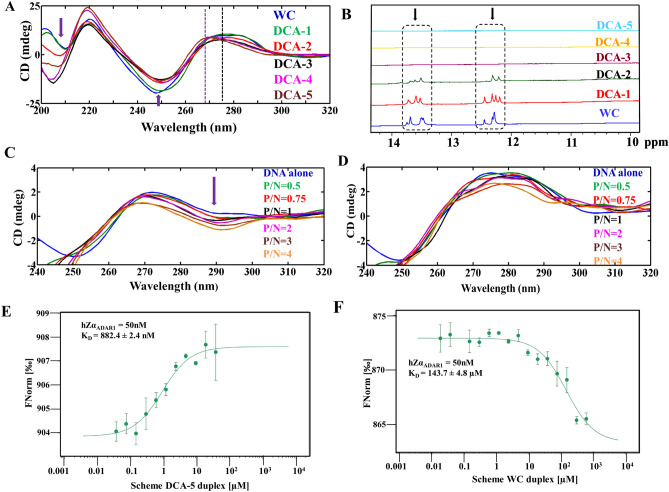
CD and NMR spectroscopic experiments reveal the increase in the extent of B–Z junction with the increasing number of A…A mismatch in CAA containing DNA sequence (Table 1). (**A)** The CD spectra corresponding to CAA containing sequences that has 1–5 A…A mismatches. As the number of A…A mismatch in the DNA duplex increases, the B–Z junction formation is also increased. This is evident from the increasing and decreasing negative peak around 205 nm and 250 nm, respectively. The shift in the positive peak (black dotted lines) towards 268 nm (purple dotted lines) is also an indication of B–Z junction formation. The MATLAB 7.11.0 software (www.mathworks.com) was used to plot the data. (**B**) Overlay of 1D proton NMR spectra of DNA duplexes that contain 1–5 A…A mismatches in the context of CAA sequence. The TopSpin 4.0.2 software (www.bruker.com) was used for processing the NMR data. (**C**) Titration of hZα_ADAR1_ with d(CAA)_5_·d(TAG)_5_ duplex that has 5 A…A mismatches (Scheme DCA-5, Table 1). The appearance of the negative peak around 290 nm as a function of increasing hZα_ADAR1_ protein concentration is a clear indication of the B–Z junction formation. In contrast, the respective negative peak is absent in the (**D**) canonical WC DNA duplex (Scheme WC). The MATLAB 7.11.0 software (www.mathworks.com) was used to plot the data. (**E**, **F**) The binding affinity between hZα_ADAR1_ and scheme DCA-5 (5 A…A mismatches) duplex measured using MST shows that the affinity between them is stronger (**E**) compared with the WC (0 A…A mismatch) DNA duplex (**F**). The law of mass action equation was used to fit the data using MO affinity software (www.nanotempertech.com) to obtain the K_D_ value.

To experimentally confirm the influence of A…A mismatch in forming the B–Z junction, we have carried out circular dichroism (CD) and 1D proton NMR experiments by considering 5 CAA DNA repeats that have A…A mismatch between 0 and 5 (Table 1). In the absence of the A…A mismatch (Table 1, WC), the characteristic B-DNA peaks (viz*.,* positive peaks around 275 nm and 205 nm, and a negative peak around 250 nm) are seen in the CD spectrum. However, when the number of A…A mismatch increases from 1 to 5 (Table 1, Schemes DCA-1 to DCA-5), the positive peak around 205 nm gradually demolishes and a negative peak emerges (Fig. 5A). Similarly, the negative peak around 250 nm gradually decreases as the number of A…A mismatches increases, which is accompanied by a shift in the positive peak from 275 to 268 nm (Fig. 5A, black and purple dotted lines). The shift in the positive peak from 275 to 268 nm, the emergence of a negative peak ~ 205 nm^34^ and a decrease in the negative peak ~ 250 nm^35^ are the characteristics of B–Z junction DNA. However, the CD spectrum corresponding to 5 A…A mismatches still retains some features of the B-DNA. For instance, despite the decrease in the negative peak around 250 nm and a shift in the positive peak from 275 to 268 nm, a negative peak that is expected around 290 nm for a Z-DNA is absent in the spectra. Nonetheless, the thermal denaturation studies exhibit a trend of biphasic melting curve (Supplementary Fig. S6). This indicates the presence of multiple conformations. In addition, the CD spectra collected at the high salt concentration (4M NaCl) for the schemes DCA-1 to DCA-5 indicate that the negative peak around 205 nm and 250 nm gradually increases and decreases respectively when the A…A mismatch increases from 1 to 5 (Supplementary Fig. S7). Together, these results represent that the CD spectra have the characteristic of both the B-DNA and Z-DNA, indicative of the presence of the B–Z junction (Fig. 5A, purple arrows).

In line with the CD results, 1D proton NMR spectra corresponding to schemes WC and DCA1 to DCA5 duplexes that have 0–5 A…A mismatches (Table 1, Schemes DCA-1 to DCA-5) exhibit a significant change in the proton peaks between 12 and 14 ppm. These peaks may correspond to an imino proton peak of the adenine and/or guanine and exhibit a reduction in the peak intensity due to the peak broadening when the number of mismatches increases. While the NMR spectra of d(CAA)·d(TTG) duplexes that have 0 (Scheme WC), 1 (Scheme DCA-1) and 2 (Scheme DCA-2) A…A mismatch(es) have the peaks between 12 and 14 ppm (Fig. 5B), the peak broadening is significant even to the extent of complete disappearance when the number of A…A mismatches increases above 2 (Fig. 5B, dashed boxes). Further, the methyl proton resonances which are insensitive to base…water proton exchange also undergo reduction in the peak intensity with respect to increasing number of A…A mismatch (Supplementary Fig. S8). Although there are some changes in the proton peaks of the 1D spectra corresponding to six duplexes (which one can expect due to the difference in the sequences), WC duplex has the highest peak intensities compared with the rest of the duplexes (for instance, ~ 1.7 ppm). This trend is in support of the reduction in the peak intensity observed in the imino proton region. As it is evident from the wavelength scan (Fig. 5A) and thermal denaturation (Supplementary Fig. S6) curve derived from the CD that the DNA sequences form the duplex, the peak broadening seen with respect to increase in the number of mismatch in 1D-NMR is the effect of A…A mismatch. Thus, it indicates that the duplex undergoes significant conformational changes when the number of A…A mismatch increases beyond 2.

#### *B–Z junction formation at the A…A mismatch site in d(CAA)*_*5*_*·d(TAG)*_*5*_* facilitates the interaction with hZα*_*ADAR1*_

The CD spectra corresponding to the titration of d(CAA)_5_·d(TAG)_5_ (Scheme DCA-5 which has 5 A…A mismatches) (N) with the hZα_ADAR1_ protein (P) show that as the concentration of hZα_ADAR1_ increases (P/N = 0–4), a new peak starts appearing around 290 nm, which is a characteristic Z-DNA peak. However, a complete inversion of the CD signal between 250 and 300 nm is not seen which is expected for a complete B to Z transition. For instance, a positive peak ~ 275 nm, a characteristic B-DNA peak, is still present with the increasing concentration of the protein. Thus, the negative peak ~ 290 nm and a positive peak ~ 275 nm indicate the presence of the B–Z junction (Fig. 5C, purple arrow). In contrast, the emergence of a negative peak around 290 nm is not seen when d(CAA)_5_·d(TTG)_5_ (Scheme WC that does not contain any A…A mismatch) is titrated with the hZα_ADAR1_ (Fig. 5D). Thus, it is clear that the A…A mismatch promotes the formation of B–Z junction which is further enhanced by the binding of hZα_ADAR1_ with d(CAA)_5_·d(TAG)_5_. Further, microscale thermophoresis experiments show that DCA-5 (882.4 nM) (5 A…A mismatches) binds strongly with hZα_ADAR1_ protein compared with WC (143.3 µM) (0 A…A mismatch) duplex (Fig. 5E,F). This further supports that the B–Z junction induced by the A…A mismatch promotes the binding with hZα_ADAR1_.

#### Exploring the conformational intermediates of the A…A mismatch using the umbrella sampling

**Figure 6:**
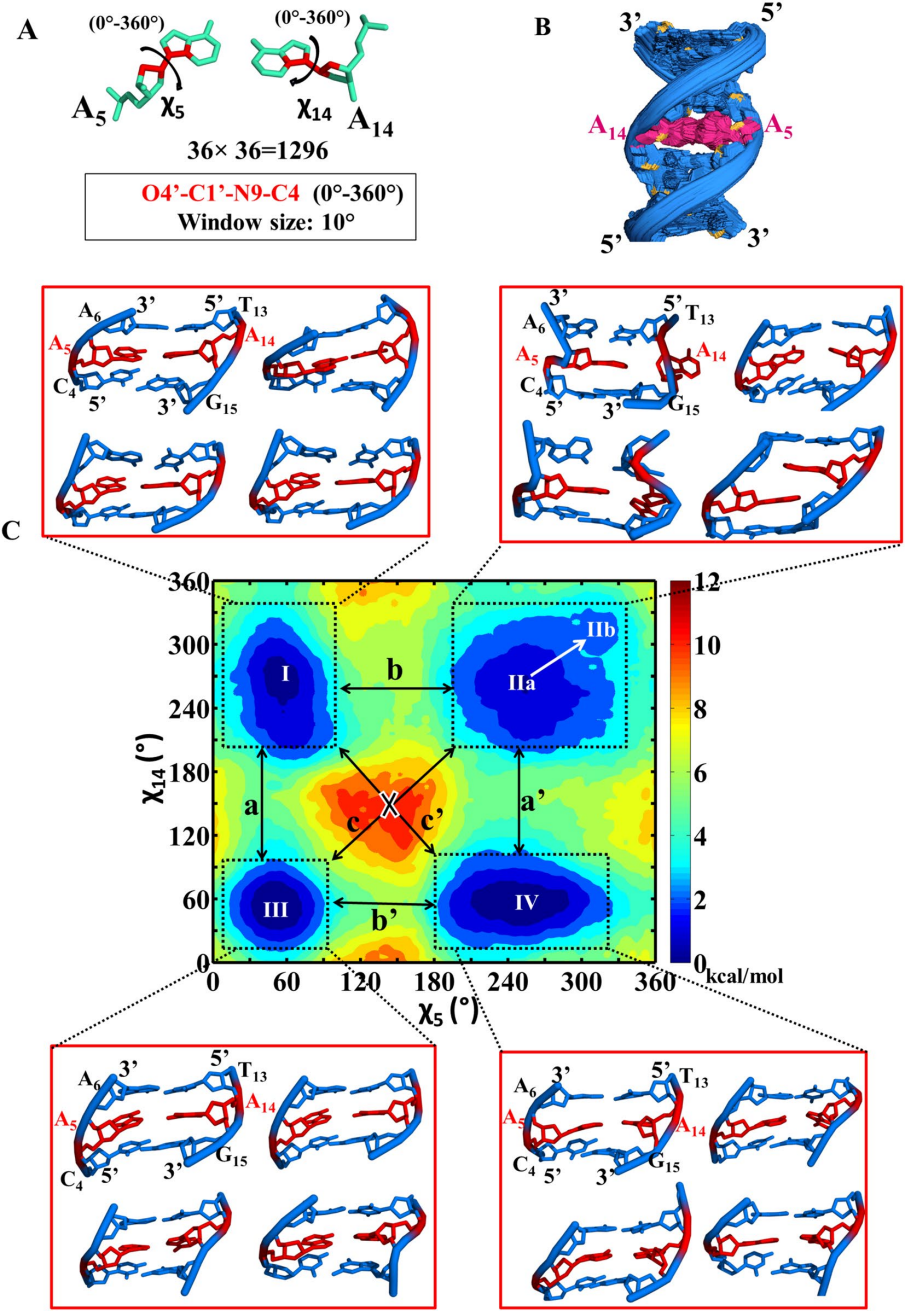
Umbrella sampling simulation corresponding to the scheme DCA-1a (Table 1) that has a single A_5_…A_14_ mismatch. (**A**) The pictorial representation of the reaction coordinates used in the umbrella sampling. Note that the *glycosyl* conformations (χ_5_ and χ_14_) corresponding to A_5_…A_14_ are sampled at 10° interval. (**B**) The superposition of 1296 starting conformations that are generated to sample the *glycosyl* conformations of A_5_ and A_14_ (36 windows each for χ_5_ and χ_14_ = 36 × 36 windows = 1296 windows) in the window size of 10°. The figures (**A**) and (**B**) were generated by using Pymol 1.3 (www.pymol.com) software^53^. (**C**) The 2D free energy map for (χ_5_, χ_14_). The labels I–IV indicates the favorable minima regions and the snapshots associated with these regions are depicted in the red color boxes. The region I corresponds to χ_5_ (20°–100°)…χ_14_ (200°–330°) and the region II corresponds to χ_5_ (190°–340°)…χ_14_ (190°–340°) (which encompasses two minima (IIa and IIb)). Similarly, regions III and IV represent the conformational spaces corresponding to χ_5_ (20°–90°)…χ_14_ (20°–90°) and χ_5_ (180°–330°)…χ_14_ (20°–100°) respectively. The possible transition between IIa (*anti…anti*) to IIb (−*syn…*−*syn*) (similar to that seen in MD, Supplementary Fig. 1A) is indicated by an arrow. The possible vertical (a and a′) and horizontal (b and b′) transitions between the minima regions are indicated by double-headed arrows and the unfavorable diagonal transitions (c and c′) between the minima regions are indicated by a cross symbol (**C**). The MATLAB 7.11.0 software (www.mathworks.com) was used to plot the data. Note that the A_5_…A_14_ mismatch is colored purple and red in (**B**) and (**C**), respectively.

**Figure 7:**
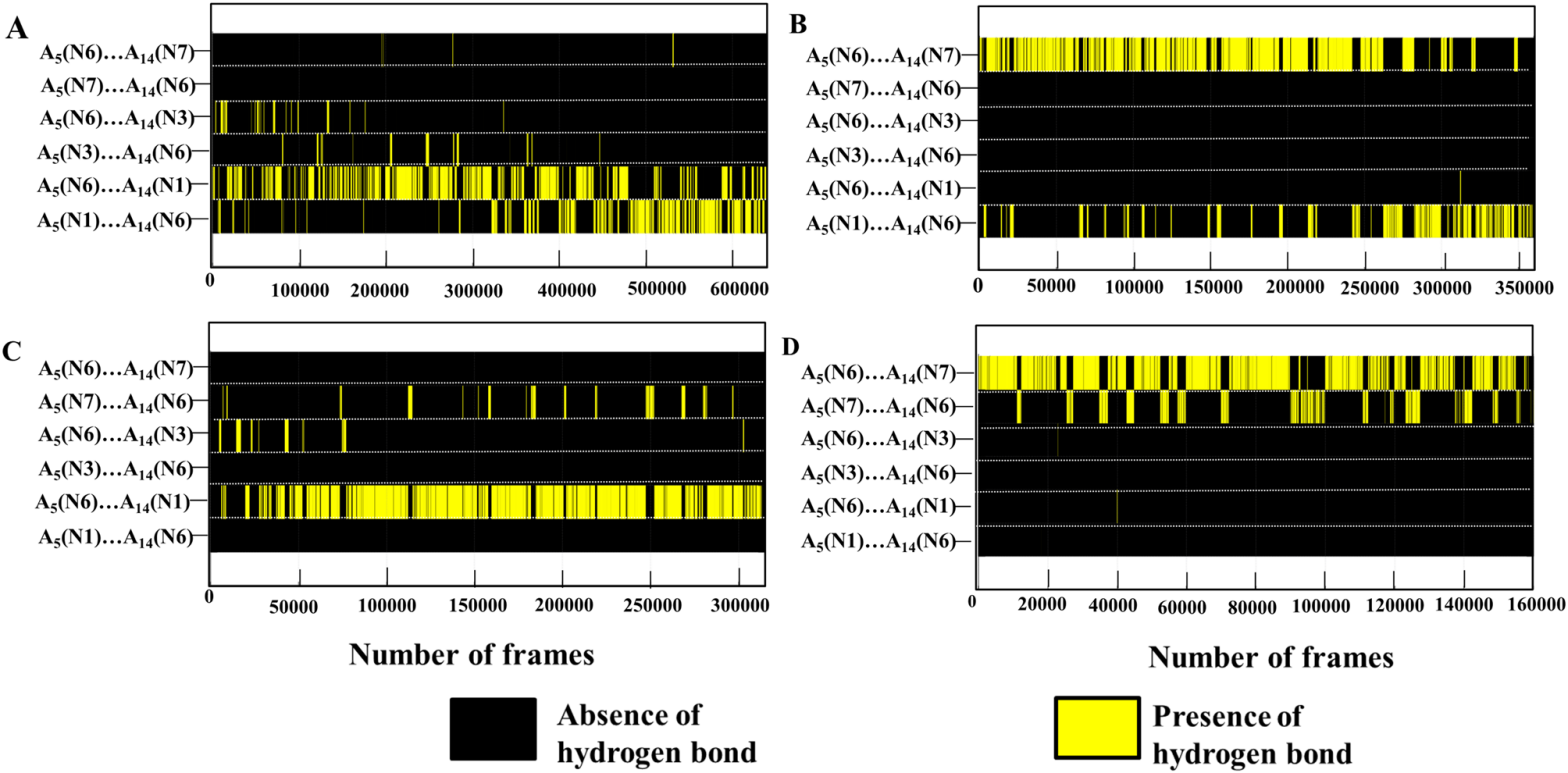
Hydrogen bond lifetime profiles corresponding to the energetically favored regions of the free energy map (dotted boxes in Fig. 6). The hydrogen bond profile representing the dynamics of A_5_…A_14_ in (**A**) *anti…anti* (χ_5_ and χ_14_ each has 16 × 16 = 256 windows in this region, wherein, each window has 2500 frames), (**B**) *anti…* + *syn,* (**C**) + *syn …anti* and (**D**) + *syn…* + *syn* regions. Note that the windows (each window has 2500 frames) corresponding to each region are arranged adjacent to each other and are represented in terms of the total number of frames along the X-axis. Refer to the text for details. The GNUPLOT 5.2 software was used to plot the data^54^.

**Figure 8:**
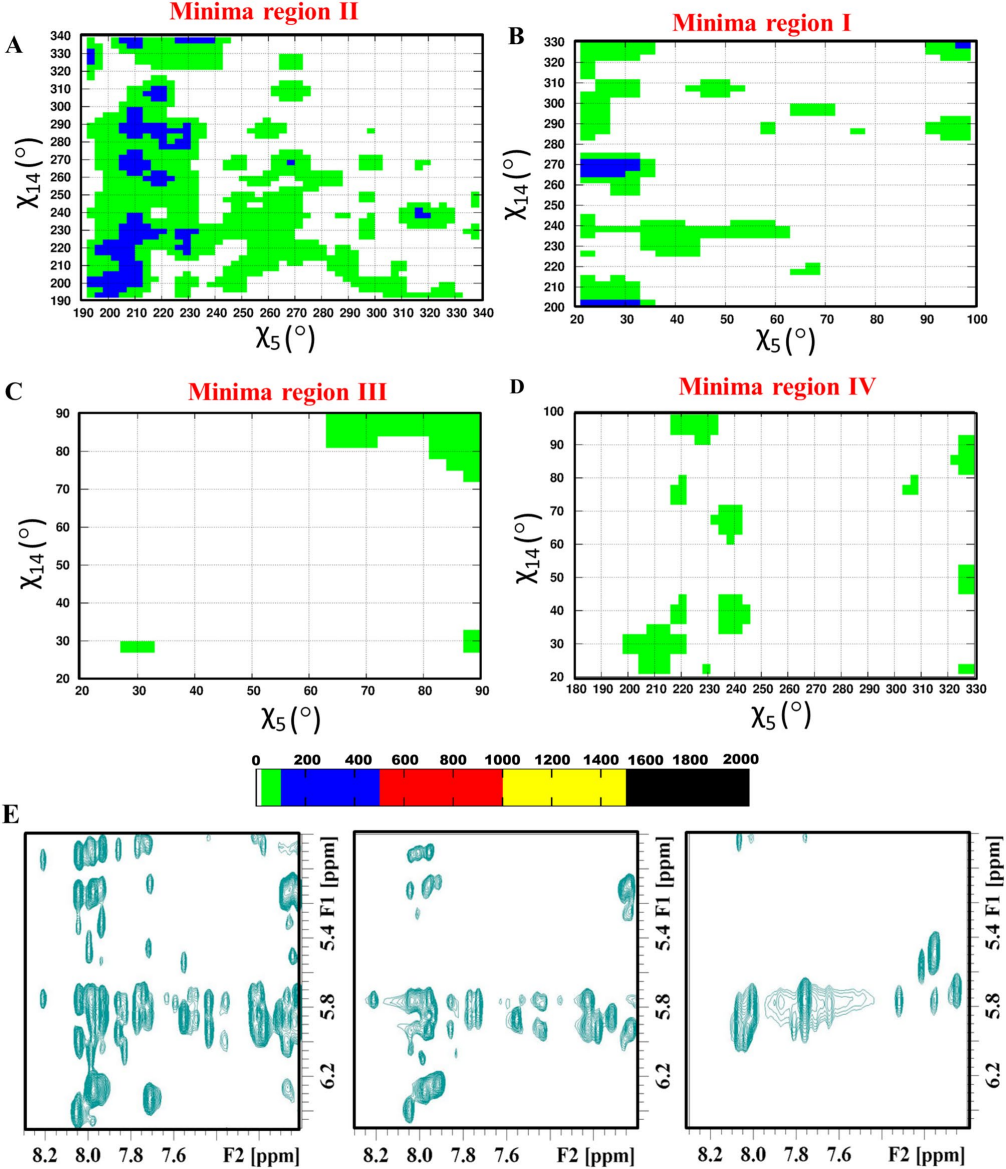
2D contour density plot illustrating the frequency of occurrence of non-hydrogen bonded A_5_…A_14_ pairs during the umbrella sampling simulation and 2D-NOESY spectra. The contour density plots are represented in terms of the frequency of occurrence (considered in the third dimension) of non-hydrogen bonded A_5_…A_14_ pairs with respect to their *glycosyl* conformational (χ_5_ and χ_14_ in X- and Y-axes respectively) preferences (dotted boxes, Fig. 6): (**A**) *anti…anti*, (**B**) + *syn…anti*, (**C**) + *syn…* + *syn* and (**D**) *anti…* + *syn*. Note that the donor…acceptor distances greater than 4 Å are considered for the plotting. The GNUPLOT 5.2 software was used to plot the data^54^. (**E**) 2D-NOESY spectra corresponding to WC (zero A…A mismatch, left), DCA-1 (one A…A mismatch, middle) and DCA-5 (five A…A mismatches, right) schemes. The TopSpin 4.0.2 software (www.bruker.com) was used for processing the NMR data.

In order to explore all the possible *glycosyl* conformational preference for the A…A mismatch, the umbrella sampling MD simulation has been carried out for the Scheme DCA-1a by considering the *glycosyl* conformations of A_5_…A_14_ as the reaction coordinates (Fig. 6A,B). The 2D potential of mean force (PMF) profile constructed from the umbrella sampling (using the last 2.5 ns of each 1296 windows) indicates that the + *syn…anti (minima I), anti…anti (minima regions IIa and IIb)*, + *syn…* + *syn (minima III)* and *anti…* + *syn (minima IV) glycosyl* conformations are energetically favored (with a free energy value of 3 kcal/mol) for the mismatched adenines (Fig. 6C) with the standard deviation not more than 0.2 kcal/mol (Supplementary Fig. S9). The PMF further indicates the possibility of transitions between 4 minima regions: two vertical transitions and two horizontal transitions. The vertical transitions are between the minima regions I and III (indicated by a) and, between minima regions II and IV (indicated by a′). The horizontal transitions are between minima regions *I* and *II* (indicated by b) and between minima regions *III* and *IV* (indicated by b′). However, the diagonal transitions, between *I* and *IV* (indicated by c) and between *II* and *III* (indicated by c′) are unfavorable due to the presence of high energy barrier between the minima regions (Fig. 6C, indicated by cross symbol). This is due to the fact that the vertical and horizontal transitions require change in the *glycosyl* conformation of just one of the mismatched adenines, whereas, the diagonal transitions require the changes in the *glycosyl* conformations of both the adenines. Interestingly, in the MD simulation, *anti…* + *syn* (minima IV)*,* + *syn…anti* (minima I) and *-syn…-syn* (minima IIb) conformations are found to be preferred (Supplementary Figs. S1A–D, S2D) over the *anti…anti glycosyl* conformation. In fact, the initial *anti…anti glycosyl* conformation moves towards −*syn…*−*syn* conformation quite early during the simulation (Supplementary Fig. S1A). Contradictorily, *anti…anti* conformation is found to be one of the favorable conformations in the umbrella sampling simulations. To our surprise, the detailed analysis indicates that the A_5_…A_14_ mismatch is highly dynamic in the *anti…anti* region compared with the other three regions (+ *syn…anti/anti…* + *syn* and + *syn…* + *syn*). The A_5_…A_14_ mismatch in this region samples a variety of base pairing schemes such as the presence or absence of N1…N6/N6…N1/N3…N6/N6…N3 hydrogen bonds, base extrusion and stacking with a shorter lifetime (Fig. 7A) compared with the other regions (Fig. 7B–D). Yet another interesting observation is that the N7…N6/N6…N7 hydrogen bond is less sampled in the *anti…anti* conformational region compared with the N1…N6/N6…N1 hydrogen bond (Fig. 7A). The + *syn…anti* region also samples N6…N3/N6…N1/N7…N6 hydrogen bonds (Fig. 7C), whereas, the *anti…* + *syn* and + *syn…* + *syn* regions sample predominantly N7…N6/N6…N7 hydrogen bond (Fig. 7B,D)*.* The N3…N6/N6…N3 hydrogen bond that evolves due to the movement of one of the mismatched adenines towards the minor groove is more visited in the *anti…anti* and + *syn…anti* regions compared with the *anti…* + *syn* and + *syn…* + *syn* regions (Fig. 7). Notably, the N3…N6/N6…N3 hydrogen bond is also seen during the MD simulation (Figs. 1B, 3B and S2B). The + *syn…* + *syn* region is more conservative compared with the other regions as it is highly confined to the N7…N6/N6…N7 hydrogen bond during the simulation (Fig. 7D). Further, A_5_…A_14_ mismatch with a total loss of hydrogen bonding interaction is quite significant in the *anti…anti* and + *syn…anti* regions (Fig. 8A,B), whereas, it is less populated in the + *syn…* + *syn* and *anti…* + *syn* regions (Fig. 8C,D). This reflects in the non-hydrogen bonded (donor…acceptor distance above 4 Å) A_5_…A_14_ mismatch population, which is significant in the *anti…anti* region (Fig. 8A). The longish hydrogen bond distances are associated with the base stacking, base pair opening and extrusion events. Such a highly dynamic nature of the A_5_…A_14_ mismatch seen in the umbrella sampling simulation is further in conformity with the 2D-NOESY spectra (Fig. 8E). The 2D-NOESY data shows that the proton-proton cross-peaks are less when the number of A…A mismatch is 5 (Scheme DCA-5) compared to the situation when the number of mismatch is 0 (Scheme WC) and 1 (Scheme DCA-1). These clearly indicate the highly dynamic nature of the mismatch.

**Figure 9:**
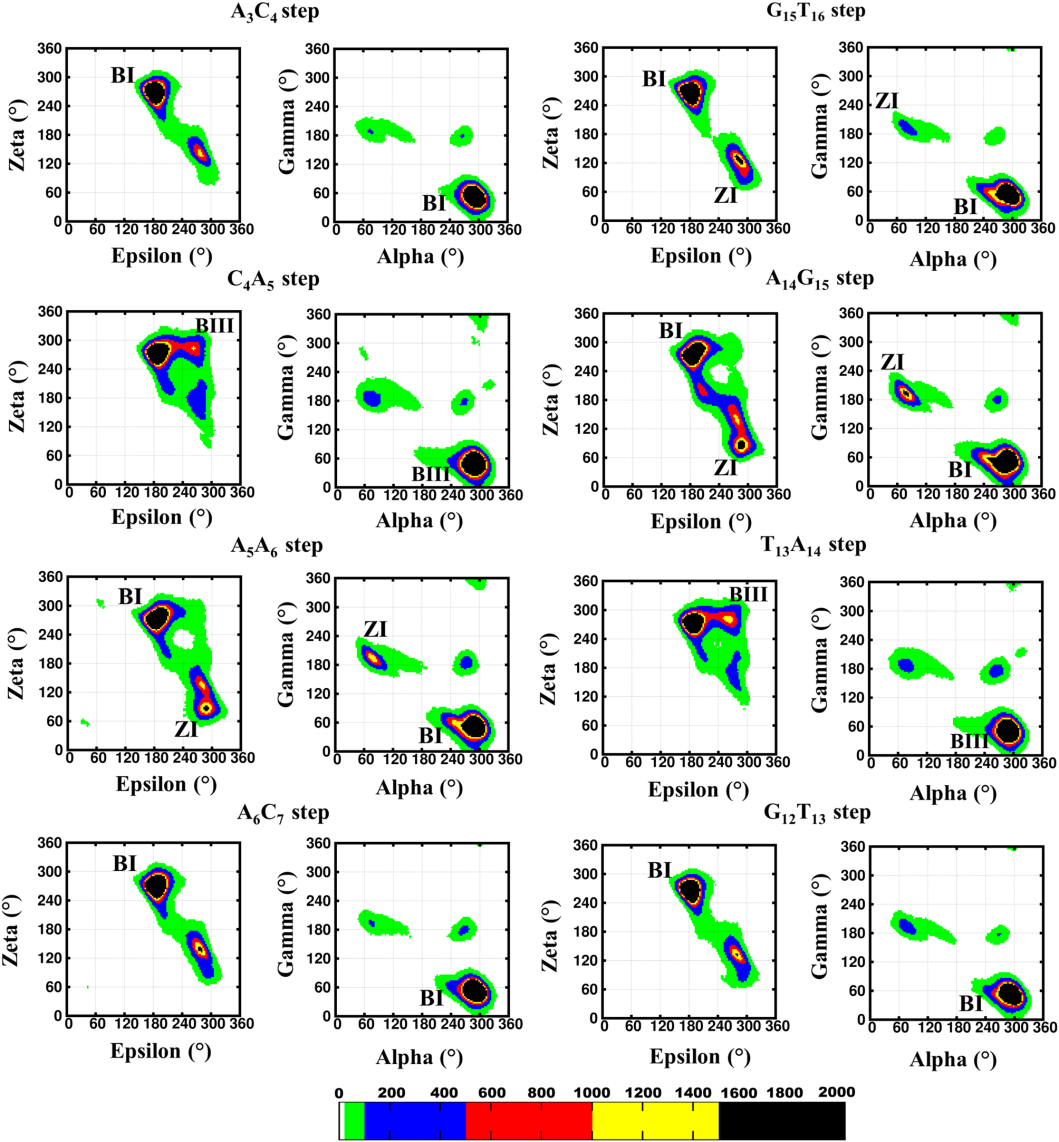
Backbone conformational preference (ε, ζ, α, γ) corresponding to the umbrella sampling simulation (Scheme DCA-1a). (ε&ζ) (1st and 3rd column) and (α&γ) (2nd and 4th column) 2D contour density plots corresponding to various steps in the vicinity of the mismatch. Note that the BI ((ε, ζ, α, γ) = (t, g^−^, g^−^, g^+^)), BII (g^−^, t, g^−^, g^+^), BIII (g^−^, g^−^, g^−^, g^+^) and ZI (g^−^, g^+^, g^+^, t) conformations are indicated adjacent to the corresponding regions. Other conformational intermediates can also be seen in the plot. The data corresponding to the last 2.5 ns simulation of the energetically favored regions (boxed in Fig. 6) is considered for the plotting. The scale corresponding to the isolines is given at the bottom. The GNUPLOT 5.2 software was used to plot the data^54^.

It is also evident from Fig. 9 that the backbone conformational angles exhibit B–Z junction characteristics at the energetically favored *glycosyl* regions (Fig. 6C). For instance, the A_5_A_6_ and A_14_G_15_ base steps take up ZI along with BI conformation. Similarly, the C_4_A_5_ and T_13_A_14_ base steps attain BIII conformation apart from the BI conformation. In addition, other intermediate conformations, which are associated with the hydrogen bond dynamics is also observed in *anti…* + *syn* (minima *IV*), + *syn…anti* (minima I) and *anti…anti* (minima II) regions (Supplementary Figs. S10–S13). Such a predominance of B–Z junction/intermediate conformational preference by (ε, ζ, α, γ) in the *anti…anti,* and + *syn…anti* (which are restrained in the umbrella sampling MD) regions may ease the movement of one of the mismatched adenines towards the minor groove to form N3…N6/N6…N3 hydrogen bond or the formation of stacked conformation at the cost of hydrogen bond to accommodate the A_5_…A_14_ mismatch in the midst of the canonical base pairs which favor B-form conformation. However, the “*syn*” *glycosyl* (a characteristic of Z-form) conformation in + *syn…* + *syn* (minima III) region can readily accommodate the N6…N7/N7…N6 hydrogen bond. The B–Z conformational preference at the mismatch site also leads to the widening of the minor groove (Supplementary Fig. S14). In any case, the dynamic nature of the A_5_…A_14_ is clear from the umbrella sampling simulations.

The umbrella sampling MD carried out to explore the effect of A_5_…A_14_ mismatch that is flanked by 5′A…T and 3′C…G (viz*.*, the reversal of CAA) in a 5′AAC/5′GTT DNA duplex (Table 1, Scheme DAC-1a) also indicates similar energetically favored (standard deviation below 0.2 kcal/mol) *glycosyl* conformations (Supplementary Figs. S15A, S16) and base pairing schemes for the mismatch (Supplementary Fig. S15B). Together, these umbrella sampling simulations reveal four equally preferable *glycosyl* conformations for the A_5_…A_14_ mismatch which is associated with a variety of spontaneous and frequent base-pairing schemes.

## Discussion

The base pair mismatches incorporated erroneously in the DNA duplex can lead to significant distortions in the DNA structure. These structural distortions act as a root cause for the concomitant biological processes. For instance, the A…A mismatch in d(CAG) and d(GAC) expansion is associated with several neuromuscular disorders^13, 14^. The current study explores the conformational dynamics of A…A mismatch that is flanked by C…G and A…T at the 5′ and 3′ respectively (Schemes DCA-1 to DCA-5 and DCA-1a) from the perspective of its recognition by the mismatch repair protein machinery to execute the DNA repair chemistry.

### B–Z junction formation at the A…A mismatch site

The MD simulations carried out here for a DNA duplex that has an A_8_…A_23_ mismatch flanked by C…G and A…T canonical base pairs on both the sides (Scheme DCA-1) indicate that the mismatch imposes significant conformational changes irrespective of the starting *anti …* + *syn/*+ *syn…anti* and *anti…anti* A_8_…A_23_ *glycosyl* conformations. The nonisostericity of A_8_…A_23_ mismatch with respect to the flanking canonical base pairs propels major conformational changes at the mismatch site: B–Z junction formation at the mismatch site (Figs. 2 and 4), formation of a N3(A)…N6(A) hydrogen bond due to the movement of one of the mismatched adenine towards the minor groove (Fig. 1D), extrusion of adenines towards the major groove (Fig. 1D, Movie S2) and adenine flipping (Fig. 1C, Movie S1). Yet another important revelation from the MD simulation is the dislike for *anti…anti glycosyl* conformation by A_8_…A_23_ mismatch and the preference for −*syn…*−*syn* and *anti …* + *syn/*+ *syn…anti glycosyl* conformations. Further, the B–Z junction formation results in the widening of the minor groove concomitant with the backbone torsion angles (ε, ζ, α, γ) preferring BIII (g^−^, g^−^, g^−^, g^+^) and ZI (g^−^, g^+^, g^+^, t) conformations. Several backbone conformations ((t, t, g^−^, g^+^), (g^−^, g^+^, g^+^, t), (g^−^, t, g^+^, t), (t, g^−^, g^+^, t), (t, t, g^−^, t) and (t, g^−^, g^−^, t)) other than that correspond to B- or Z- forms are also observed (Figs. 2 and 4) due to the spontaneous exchange between different A_8_…A_23_ base pairing schemes. A similar B–Z junction characteristic is also observed when the A…A mismatch is flanked by C…G and G…C baser pairs^19,20^. It is noteworthy that despite such backbone conformational preferences, the sugar pucker predominantly prefer C2′-endo although a minor population of C3′-endo is seen when the adenine takes up *anti* conformation (Supplementary Fig. S17A,B).

### The frequent exchange between the A…A base pair intermediates during the umbrella sampling

The umbrella sampling simulations indicate that the *anti…anti, anti…* + *syn, *+ *syn…anti* and + *syn…* + *syn glycosyl* conformations are favored by the A_5_…A_14_ mismatch (Scheme DCA-1a) (Fig. 6C). As mentioned above, the A_5_…A_14_ mismatch samples a variety of conformations such as base stacking, base extrusion, base pair opening and minor groove widening, etc*.* along with aberrant BIII and ZI conformations for (ε, ζ, α, γ). It is found that + *syn…* + *syn glycosyl* conformation prefers N6…N7/N7…N6 hydrogen bond. Further, + *syn…* + *syn glycosyl* conformational region prefers south type puckering for the deoxyribose sugar, whereas, the *anti…anti* conformational region samples a minor population of north type sugar pucker in addition to the south type pucker (Supplementary Fig. S17C–F). The *anti…anti* region encounters a frequent transition between the adenine extrusion, adenines stacking and the hydrogen bonds, N1…N6/N6…N1, N7…N6/N7…N6 and N3…N6/N6…N3 (Fig. 7A, Movie S3), (Fig. 6C, Movie S4). These conformations are relatively short-lived compared with the conformations observed in + *syn…* + *syn glycosyl* conformational region (Fig. 7D). Such conformational dynamics is also seen in *anti…* + *syn/*+ *syn…anti* regions (Fig. 7B,C). Thus, it is clear that the B–Z junction favored by the A_5_…A_14_ mismatch (Movie S5) may lower the energy penalty for the mismatch to sample a variety of energetically favored and short-lived mismatch conformations. Further, the PMF shows the following enegetically favorable transitions, indicating the dynamic nature of the mismatch (Fig. 6C): + *syn…anti* ⇔ + *syn…* + *syn*, *anti…anti* ⇔ *anti…* + *syn, * + *syn…anti* ⇔ *anti…anti* and + *syn…* + *syn* ⇔ *anti…* + *syn.* Concomitantly, the 2D-NOESY experiments also indicate that the number of cross-peaks diminishes (indicating the frequent movement) with the increasing number of A…A mismatch (Fig. 8E).

Interestingly, the N1…N6 hydrogen bond^18^ as well as the N3…N6 hydrogen bond facilitated by the extrahelical movement of one of the adenines towards the minor groove^36^ are seen in the earlier NMR investigations. Further, the complex of *E. coli* mismatch repair protein with the DNA shows the widening of the minor groove at the mismatch site (PDB ID: 2WTU). As the A…A mismatch spontaneously leads to aberrant B–Z junction which widens the minor groove and facilitate the sampling of a variety of A…A mismatch conformations (Fig. 6C), it may ease the recognition of the mismatch site by the repair proteins. Further, the minor groove extrahelical conformation (forming N3…N6/N6…N3 hydrogen bonds) may also act as a trapping point. For instance, the crystal structure of the *E. coli* mismatch repair protein MutS and DNA duplex (having a A…A mismatch) complex shows that the N6 of one the adenines moves towards the minor groove and interacts with the protein (Supplementary Fig. S18). Thus, this supports that the protein may recognize the A…A mismatch through the N6 of one of the adenines at the minor groove side.

### The number of A…A mismatch increases the degree of B–Z junction in the CAA sequence

CD investigations carried out to prove that A…A mismatch in the context of CAA induces B–Z junction reveals that as the number of A…A mismatch increases (2–5) in the DNA duplex (Schemes DCA-2 to DCA-5), the extent of B–Z junction also increases. This is evident from the increase and decrease in the negative peak intensity ~ 205^34^ and 250 nm respectively^35,37^ (Fig. 5A). Nonetheless, the canonical W&C duplex (Scheme WC) does not exhibit these signature peaks implicating the non-existence of the B–Z junction in the same (Fig. 5A, blue line). In accordance with the CD results, 1D proton NMR spectra of the DNA duplexes that possess 0–5 A…A mismatch(es) show the peak broadening (to the level of disappearance) in between 12 ppm and 14 ppm (Fig. 5B). This is indicative of significant structural changes in the duplex with respect to the increase in the number of A…A mismatch. This is also further supported by the diminishing cross-peaks in the 2D NOESY (Fig. 8E). These show the dynamic nature of the A…A mismatch as observed in MD and umbrella sampling MD. A similar disappearance of proton peaks between 12 and 14 ppm is also seen in the 1D NMR spectra of d(GAC)_7_·d(GAC)_7_ DNA duplex (that has 7 A…A mismatches)^20^.

### A…A mismatch facilitates the binding of d(CAA)_5_·d(TAG)_5_ with hZα_ADAR1_

The titration of d(CAA)_5_·d(TAG)_5_ (Scheme DCA-5) with the hZα_ADAR1_ indicates through the appearance of a negative peak ~ 290 nm that the duplex is being recognized by the protein (Fig. 5A). However, the complete conversion of B-form to Z-form is not observed as seen in the case of d(GAC)_7_·d(GAC)_7_^20^. This is perhaps due to the fact that the latter has a CG step which is absent in the former. It has also been shown in the previous studies that hZα_ADAR1_ recognizes CG steps to recognize the duplex^38^. Thus, the presence of A…A mismatch (that prefers B–Z junction) along with the CG step lead to the complete conversion to Z-form in d(GAC)_7_·d(GAC)_7_. Nonetheless, the lone presence of A…A mismatch in the former simply leads to B–Z junction. Thus, the complete inversion of CD spectra observed in the case of d(GAC)_7_·d(GAC)_7_ upon titration with hZα_ADAR1_ is not seen in d(CAA)_5_·d(TAG)_5_ (Fig. 5C). However, the increase in the extent of a negative peak ~ 290 nm upon titration with hZα_ADAR1_ is an indication of B–Z junction formation^39,40^. Further, such a B–Z junction formation is not observed in the CD spectra of canonical d(CAA)_5_·d(TTG)_5_ duplex (Scheme WC) when it is titrated with the hZα_ADAR1_ (Fig. 5D). Thus, the increase in the extent of B–Z junction with respect to the increasing number of A…A mismatch in the context of CAA sequence (Fig. 5A) as well as the ability of hZα_ADAR1_ to recognize the d(CAA)_5_·d(TAG)_5_ duplex (that has 5 A…A mismatches) indicate that the protein traps the preformed B–Z junctions in the duplex for the binding.

## Conclusion

The detection and elimination of the falsely formed non-canonical base pairs during the replication is a crucial process. The mismatch repair proteins detect the non-canonical base pairs and repair the system. In this context, the conformational dynamics induced by the A…A mismatch in the midst of a d(CAA)·d(TAG) sequence is explored here. MD and umbrella sampling MD results presented here clearly state that the A…A mismatch favors the aberrant B–Z junction that offers less energy penalty to sample a variety of mismatch base pairing schemes. The sampled base pairing conformations are, the extrusion of adenine(s) towards the major/minor groove, adenine flipping, adenines stacking and a number of hydrogen bonding schemes, which are short-lived. A similar characteristic is also seen for A…A mismatch in the context of d(AAC)·d(GAT) sequence. The 2D-NOESY experiment also indicates the highly dynamic nature of the A…A mismatch as the number of cross-peaks diminish with the increasing number of A…A mismatch. The extra-helical movements of adenines toward the grooves, specifically toward the minor groove which facilitate the N3…N6 hydrogen bond (exposes one of A’s in the minor groove), may act as a trapping point for the mismatch repair proteins such as MSH2, MSH3 and MSH6 to perform the enzymatic reaction. CD and NMR reveal that the increasing number of A…A in the context of CAA sequence increases the extent of the B–Z junction in the duplex. Thus, the B–Z junction formation at the A…A mismatch site circumvents the mechanistic effect of base pair nonisostercity with the flanking canonical base pairs. The concomitant base pair dynamics may further ease the accessibility of the A…A mismatch to the repair proteins.

## Methods

### MD simulation setup

The starting models for the 15mer d(CAA) (Schemes DCA-1, Table 1) were constructed using 3DNuS web server^41^. Based on the previous experimental studies on A…A mismatch containing RNA duplex^28,29^, two different starting models (*anti…anti* and *anti…* + *syn/*+ *syn…anti glycosyl c*onformations for the A…A mismatch) were considered for the simulations. Using the former as the template, the latter were generated using XPLOR-NIH^42^ as discussed elsewhere^19,20^. Subsequently, these models were solvated in a TIP3P water box and net neutralized with the Na^+^ ions by using the LEaP module of AMBER 12 suite^43^. Following 70 ps equilibration at 300 K, the production run was extended to 500 ns individually for both the models^19,20^. The production run was carried out at the isobaric and isothermal conditions (NPT) with a 2 fs integration time scale. A cut-off distance of 10 Å was used for non-bonded interactions. The FF99SB (parm99 without correction) force field was used in the simulations.

### Umbrella sampling simulation setup

The conformational space accessible to the A…A mismatch *glycosyl* dihedral angles was sampled using the umbrella sampling MD simulations. The *glycosyl* dihedral angles (*chi)* corresponding to both the adenines in the A_5_…A_14_ mismatch were used as the reaction coordinates (Scheme DCA-1a). The *glycosyl* dihedral angles of each adenine were sampled using 36 windows between 0° to 360° with 10° interval. Thus, a total of 36 × 36 = 1296 windows were sampled, for which, the starting models with the appropriate *glycosyl* conformations were generated using XPLOR-NIH^42^. For the umbrella sampling, only the central 9mer sequence (Scheme DCA-1) was considered, wherein, the central CAA having the A_5_…A_14_ mismatch in the middle was flanked by a canonical CAA triplet on both the sides. In addition, the umbrella sampling was also carried out for 5′AAC, wherein, A_5_…A_14_ mismatch was flanked by 5′-A…T and 3′-C…G respectively (Scheme DAC-1a).

All the 1296 starting conformations with the appropriate *glycosyl* dihedral angles were solvated in a TIP3P water box and net neutralized with the Na^+^ ions. Each conformation was preceded with an equilibration followed by the production run. The equilibration protocol was carried out in several steps as explained in the earlier studies^44–47^ but, with an added positional restraint with a force constant of 100 kcal/mol rad^2^ on both the mismatched adenines. This step was carried out for 140 picoseconds in order to remove the steric hindrance in the model. The production run was extended up to 3 ns for each conformation at the isobaric and isothermal conditions (NPT). A restraint potential force constant of 100 kcal/mol rad^2^ was imposed on both the *glycosyl* dihedral angles of the mismatched adenines during the production run. The PMEMD module of AMBER 16 suit^48^ with the FF99SB (parm99 without correction) force field was used for the simulations. A 2 fs integration time and a 10 Å cut-off distance for non-bonded interactions were used during the simulation. The weighted histogram analysis method (WHAM)^49^ was used for generating the 2D potential of mean force (PMF) profile from the last 2.5 ns trajectories. The error estimation was calculated by constructing individual PMFs for 5 (0.5–1 ns, 1–1.5 ns, 1.5–2 ns, 2–2.5 ns and 2.5–3 ns) as well as 3 (0.5–1 ns, 0.5–2 ns and 0.5–3 ns) time blocks. Finally, the standard deviation among the PMFs (separately for 5- and 3-time blocks) was calculated.

*Cpptraj* module of Amber 16 was used to post-process the MD and the umbrella sampling MD trajectories as well as to calculate the hydrogen bond distance and RMSD^50^. Sugar pucker, *glycosyl* and backbone conformation angles were extracted from the output of 3DNA using in-house scripts^51^. VMD^52^ and Pymol^53^ were used for the visualization of trajectories. MATLAB 7.11.0 (www.mathworks.com) and GNUPLOT 5.2^54^ software packages were used for plotting the graphs. The individual snapshots corresponding to every frame of the trajectory was created using VMD^52^ and the movie was generated through videoMach plugin software 5.15.1 (www.gromada.com/videomach/).

### Purification of hZα_ADAR1_

*E. coli* BL21 (DE3) bacterial cells were used for the expression and purification of hZα_ADAR1_ using the protocol described in the earlier studies^20, 55^. The protein concentration was measured spectroscopically with an extinction coefficient of 8480 M^−1^ cm^−1^ at 280 nm.

### Sample preparation

DNA oligonucleotides (Table 1) with HPLC grade were purchased from Bioserve. The duplexes were prepared by denaturing the complementary oligonucleotides (Table 1) at 95 °C for 10 min followed by a room temperature cooling for 3 h in 10 mM phosphate buffer and 10 mM NaCl (pH 7.4). The DNA duplex…hZα_ADAR1_ complex for CD experiments were prepared by increasing the hZα_ADAR1_ concentration while retaining the DNA duplex concentration. The complex was prepared by fractional addition of the hZα_ADAR1_ protein to the DNA followed by the incubation of 1 h at 25 °C.

### CD spectroscopy

All the CD experiments were carried out in JASCO-1500 at 25 °C in the wavelength range of 190–320 nm. The data were collected in triplicate and the baseline correction was done with an appropriate buffer. For the DNA duplex…hZα_ADAR1_ titration, the protein (P)/nucleic acids (N) ratios of 0, 0.50, 0.75, 1:1, 1:2, 1:3 and 1:4 were used by keeping the DNA concentration as a constant (40 μM). The CD data was analyzed through spectra manager software (www.jascoinc.com) and verified with the reference dataset of CD-NuSS webserver^56^.

### NMR experiments

All the NMR experiments were collected in a 700 MHz instrument equipped with a 5 mm TCI H-C/N-D Cryoprobe. 1.2 mM concentration of DNA duplexes that have A…A mismatches in the range of 0–5 (Schemes WC, DCA1 to DCA5) were used. DNA duplexes were prepared in the NMR buffer (10% D_2_O, 10 mM sodium phosphate, 10 mM NaCl and pH 7.4). All the NMR experiments were performed at 25 °C. The 1H pulse calibration was done by complete nutation of 360° and then, the actual 90° pulse width was obtained. Water presaturation is obtained by finding the exact position of the water signal and applied continuous pulse during the recycle delay. 1D proton NMR was obtained with 256 scans with water presaturation.

2D NOESY data was acquired using phase-sensitive NOESY with water presaturation for the Schemes DCA-1, DCA-5 and WC. The NOESY experiment was recorded with 16 scans with 2048 time domain points in the direct dimension and 256 points in the indirect dimension. The data was recorded for the NOE mixing times of 80, 150, and 300 ms to check the cross peak intensity. The acquired 1D and 2D data were processed and analyzed using TopSpin 4.0.2 NMR software (www.bruker.com).

### Microscale thermophoresis

The binding affinity between hZα_ADAR1_ and DCA-5/WC duplexes was estimated using microscale thermophoresis (MST) assay. For this assay, the His-tagged hZα_ADAR1_ protein (His_6_-GB1-hZα_ADAR1_) was labeled with RED-tris-NTA dye (His-Tag labeling kit) as per the labeling procedure mentioned in the kit (Cat# MO-L008). The binding assay was performed by titrating 50 nM concentration of labeled hZα_ADAR1_ protein with unlabeled DNA duplex which was diluted serially (from 600 to 0.018 µM). 10 mM NaCl and 10 mM phosphate buffer (pH 7.4) was used for the assay. Following the incubation for 30 min at 25 °C, all the samples were loaded in MST-standard treated capillaries. The dissociation constant (K_D_) measurement was performed in triplicate using 40% LED power and 40% MST power in NanoTemper monolith instrument NT.115 at 25 °C. The law of mass action equation was used to fit the data using MO affinity software to obtain the K_D_ value.

## Supplementary Information

Supplementary Information 1.

Supplementary Video S1.

Supplementary Video S2.

Supplementary Video S3.

Supplementary Video S4.

Supplementary Video S5.
