# Supplementary material for: Spontaneous and frequent conformational dynamics induced by A…A mismatch in d(CAA)·d(TAG) duplex
Source: Sci Rep. 2021 Feb 11;11:3689. doi: 10.1038/s41598-021-82669-4 (PMC7878774; doi:10.1038/s41598-021-82669-4)
Supplement: Supplementary file 1 — Supplementary Information 1. [file 41598_2021_82669_MOESM1_ESM.docx]

Supplementary Information

**Spontaneous and frequent conformational dynamics induced by A…A mismatch in d(CAA).d(TAG) duplex**

Yogeeshwar Ajjugal^1^, Kripi Tomar^1,#^, D Krishna Rao^2,#^ and Thenmalarchelvi Rathinavelan^1^*

^1^Department of Biotechnology, Indian Institute of Technology Hyderabad, Kandi, Sangareddy District, Telangana State-502285, India.

^2^Tata Institute of Fundamental Research 36/P, Gopanpally Mandal, Ranga Reddy District, Hyderabad, Telangana State-500107, India.

^#^Equal contribution

*For correspondence: tr@iith.ac.in

Running title

Nonisomorphism causes frequent conformational rearrangement at the A…A mismatch site

Keywords: A…A mismatch, A…A flipping, A…A stacking, B-Z junction, hZα_ADAR1_, molecular dynamics simulation, umbrella sampling, circular dichroism, NMR

**Supplementary Figures**

**Movie S1:** Movie showing the A_8_ flipping during 500ns simulation (Scheme DCA-1). Note that the A_8_…A_23_ mismatch is modeled to have *anti…anti* starting *glycosyl* conformation. The individual snapshots corresponding to every frame of the trajectory was created using VMD [1] and the movie was generated through videoMach plugin software 5.15.1 ([www.gromada.com/videomach/](http://www.gromada.com/videomach/)).

**Movie S2:** Movie showing the A_8_ extrusion during the 500ns simulation (Scheme DCA-1). Note that the A_8_…A_23_ mismatch is modeled to have *anti…anti* starting *glycosyl* conformation.

**Movie S3:** Movie showing the dynamic nature of the A_5_…A_14_ hydrogen bond observed during the umbrella sampling simulation (Scheme DCA-1a).

**Movie S4:** Movie showing the stacking of A_5_ & A_14_ during the umbrella sampling simulation (Scheme DCA-1a).

**Movie S5:** Movie showing the B-Z junction formation around the A_5_…A_14_ mismatch site during the umbrella sampling simulation (Scheme DCA-1a).

| **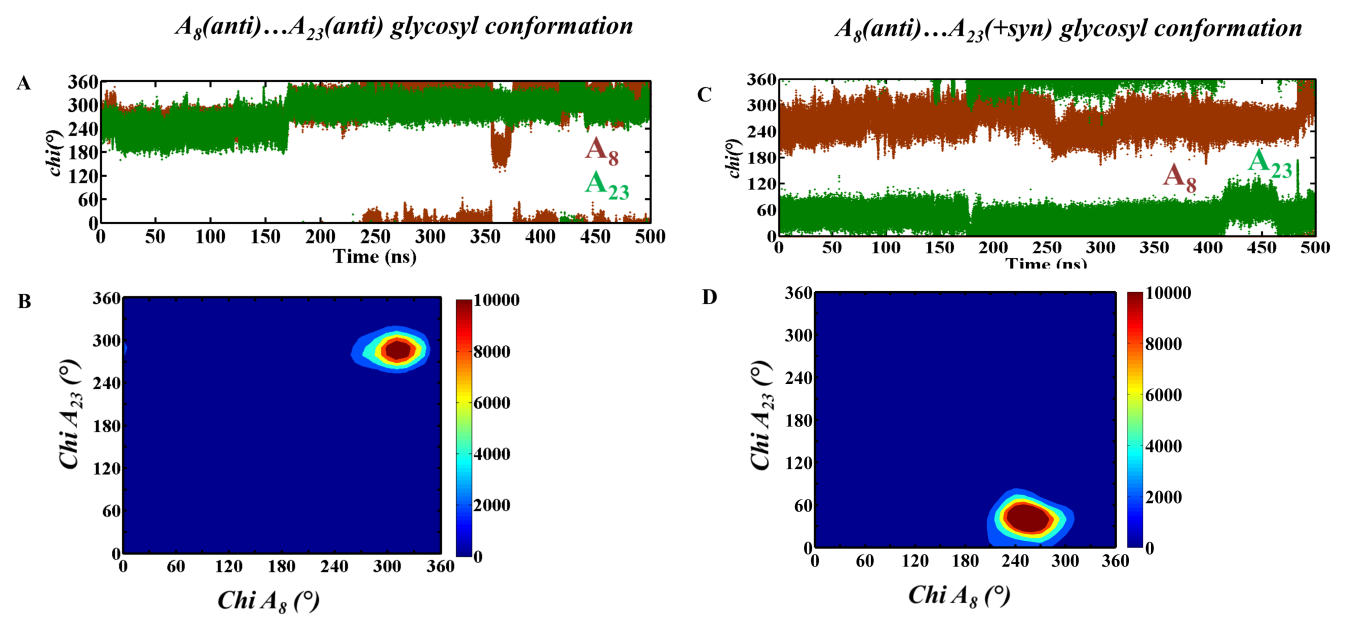** |
| --- |
| **Figure S1. The fluctuations in A_8_ & A_23_** ***glycosyl* conformations observed during the MD simulation of the scheme DCA-1 (Table 1).** Time vs *chi* profile **(A&C)** and the contour density plot **(B&D)** corresponding to *anti…anti* **(A&B)** & *anti…+syn* **(C&D)** starting A_8_&A_23_ *glycosyl* conformations. The scale corresponding to the isolines (reflecting the number of occurrences) is given alongside the contour plot (**B&D**). The MATLAB 7.11.0 software (www.mathworks.com) was used to plot the data. |

| **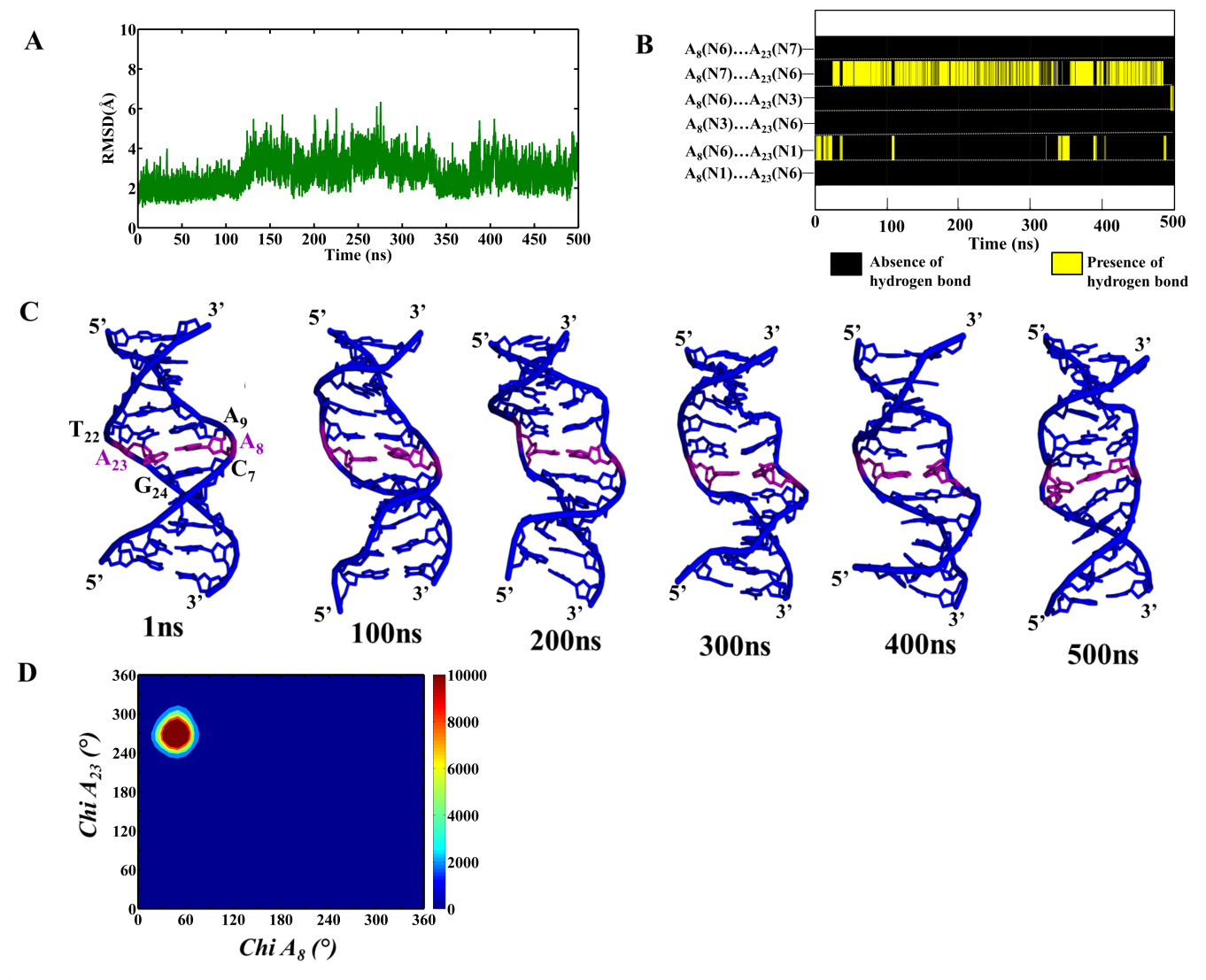** |
| --- |
| **Figure S2. Analysis of DCA-1 DNA duplex that has *+syn*…*anti* starting *glycosyl* conformation for A_8_…A_23_. (**A) Time vs RMSD profile. (B) Life time (X-axis) of different A_8_…A_23_ hydrogen bond schemes (Y-axis). The GNUPLOT 5.2 software was used to plot the data [2]. (C) Snapshots showing the local B-Z junction formation at the mismatch site. This figure was rendered by using pymol 1.3 software (www.pymol.com). (D) The contour density plot corresponding to mismatch *glycosyl* conformations. The scale corresponding to the isolines (reflecting the number of occurrences) is given alongside the contour plot. Note: A_8_…A_23_ mismatch is represented in purple color in the cartoon representation. The MATLAB 7.11.0 software (www.mathworks.com) was used to plot the data. |

| **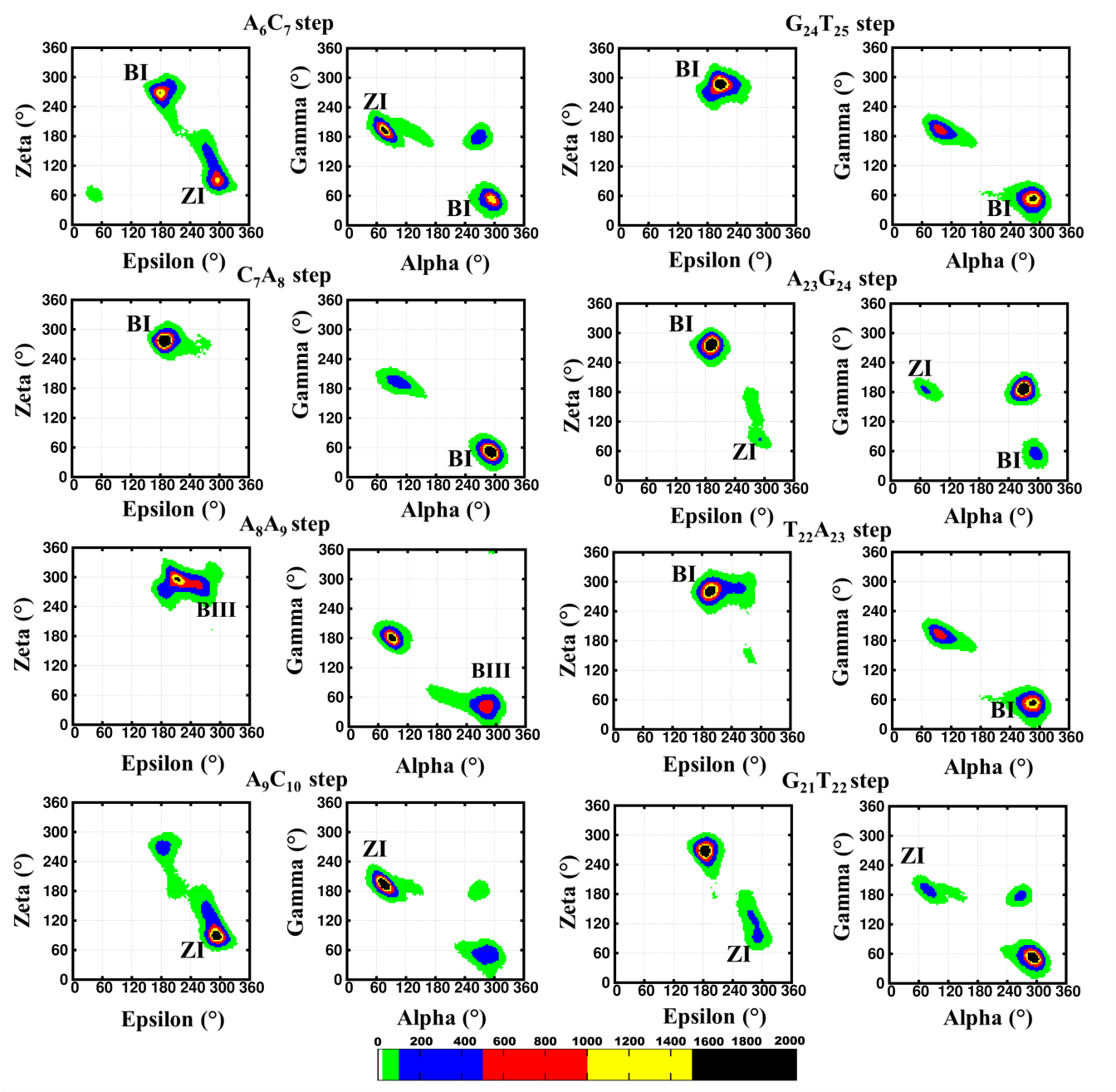** |
| --- |
| **Figure S3. The backbone torsion angles (**ε,ζ,α,γ**) corresponding to the central pentamer that encompass A_8_*(+syn)...A_23_(anti)* mismatch (scheme DCA-1).** (ε&ζ) (1^st^ and 3^rd^ column) and (α&γ) (2^nd^ and 4^th^ column) 2D contour density plots corresponding to various steps in the vicinity of the mismatch. Note that the BI ((ε,ζ,α,γ)=(t,g^-^,g^-^,g^+^)), BII (g^-^,t,g^-^,g^+^), BIII (g^-^,g^-^,g^-^,g^+^) and ZI (g^-^,g^+^,g^+^,t) conformations are indicated adjacent to the corresponding regions. Other conformational intermediates can also be seen in the plot. The trajectories corresponding to the last 300ns simulation is considered for the plotting. The scale corresponding to the isolines is given at the bottom. The GNUPLOT 5.2 software was used to plot the data [2]. |

| **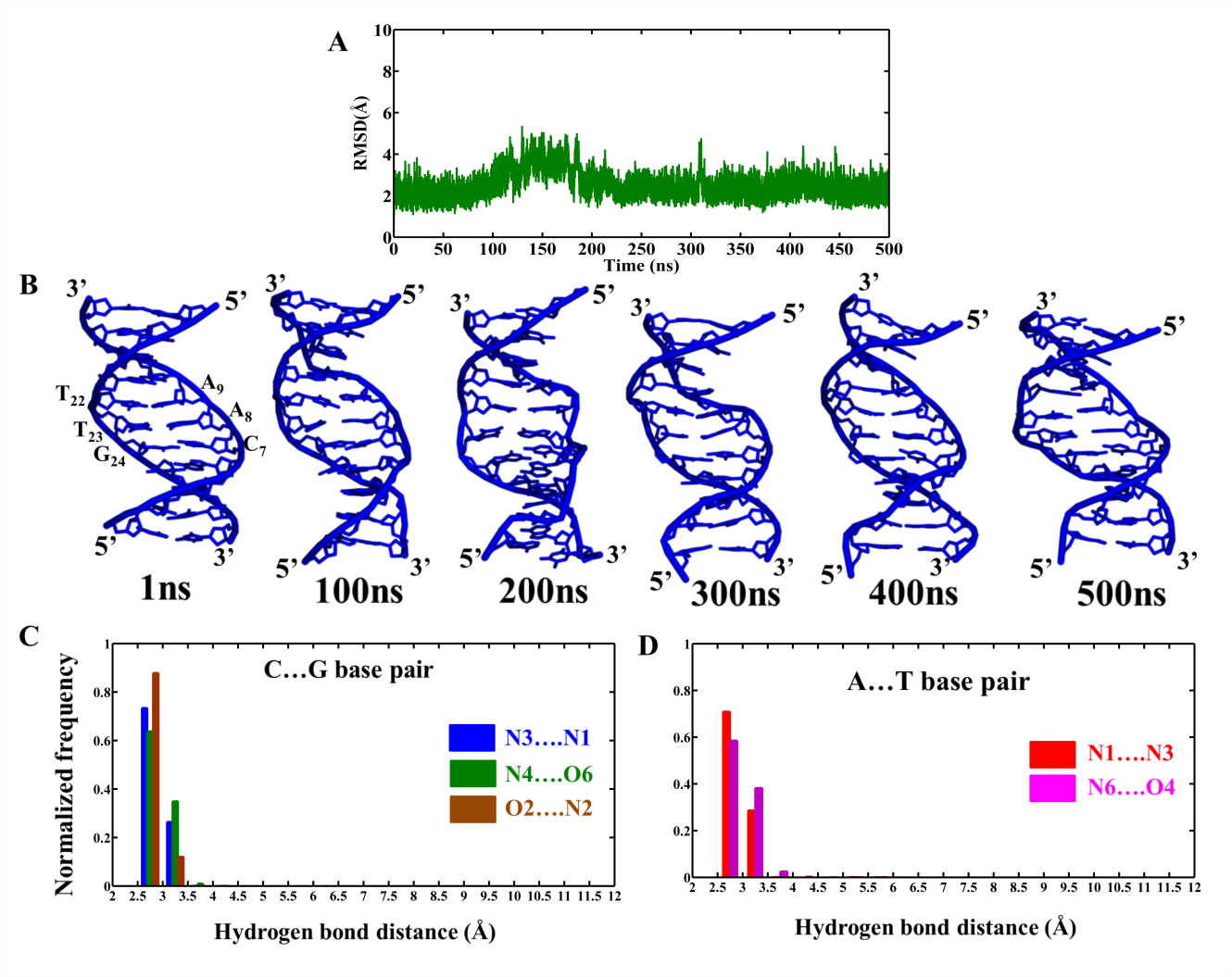** |
| --- |
| **Figure S4. Retention of B-form geometry during the MD simulation of the control duplex (Scheme WC).** (A) Time vs RMSD profile. (B) Snapshots corresponding to different time intervals of the MD simulation resemble the B-form conformation. This figure was generated by using pymol 1.3 (www.pymol.com). (C and D) Histogram corresponding to the hydrogen bond distances of G…C (C) & A…T (D) base pairs indicates that the distance falls in the range of 2.5Å-3.5Å. Note: The figures A, C&D were plotted by using MATLAB 7.11.0 software (www.mathworks.com). |

| **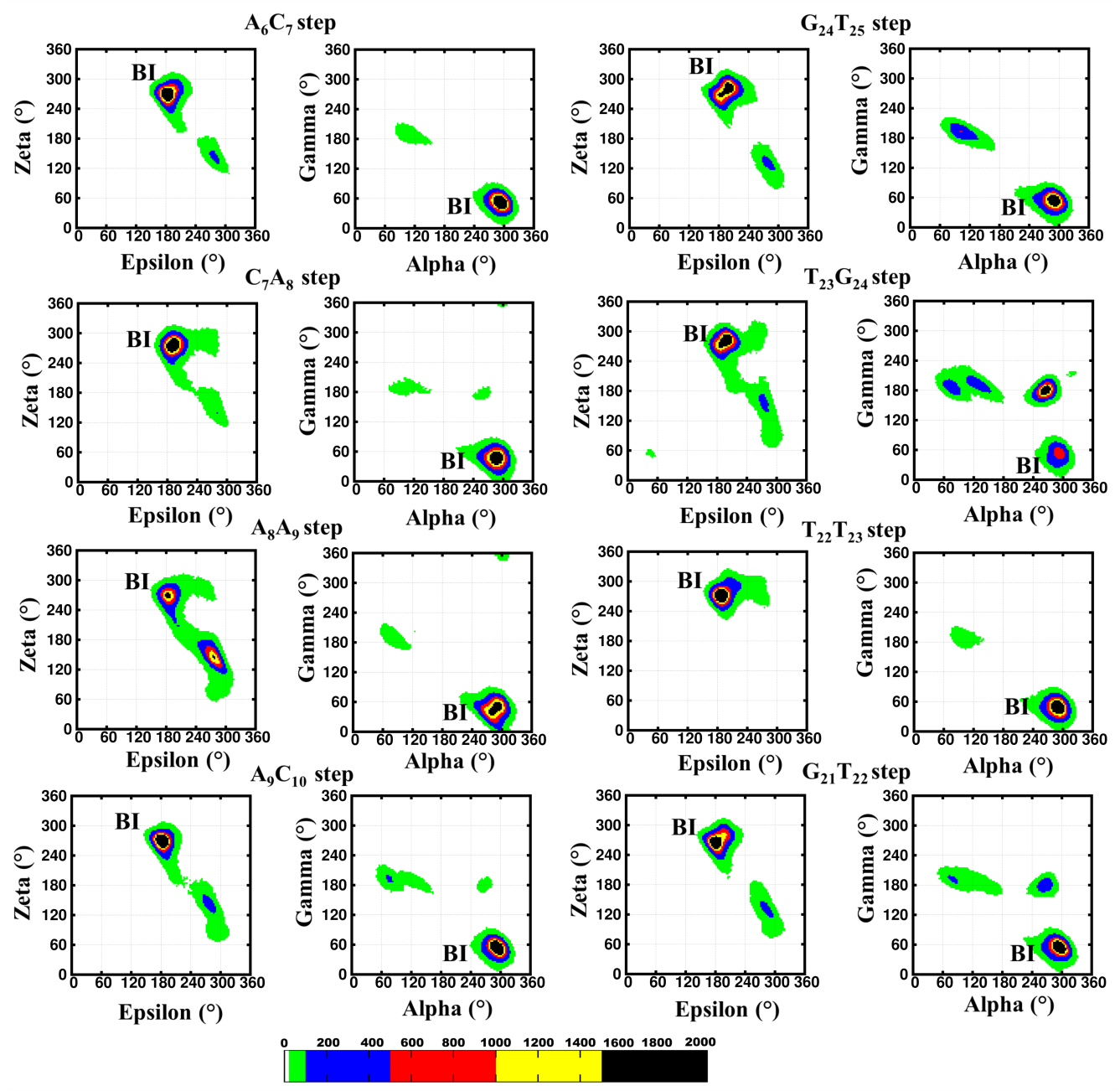** |
| --- |
| **Figure S5. The backbone torsion angles (**ε,ζ,α,γ**) corresponding to the central pentamer of the scheme WC which has only the canonical base pairs.** (ε&ζ) (1^st^ and 3^rd^ column) and (α&γ) (2^nd^ and 4^th^ column) 2D contour density plots corresponding to various steps in the vicinity of the mismatch. Note that the BI ((ε,ζ,α,γ)=(t,g^-^,g^-^,g^+^)), BII (g^-^,t,g^-^,g^+^), BIII (g^-^,g^-^,g^-^,g^+^) and ZI (g^-^,g^+^,g^+^,t) conformations are indicated adjacent to the corresponding regions. Note the dominance of B-form geometry. The trajectories corresponding to the last 300ns simulation is considered for the plotting. The scale corresponding to the isolines is given at the bottom. The GNUPLOT 5.2 software was used to plot the data [2]. |

| **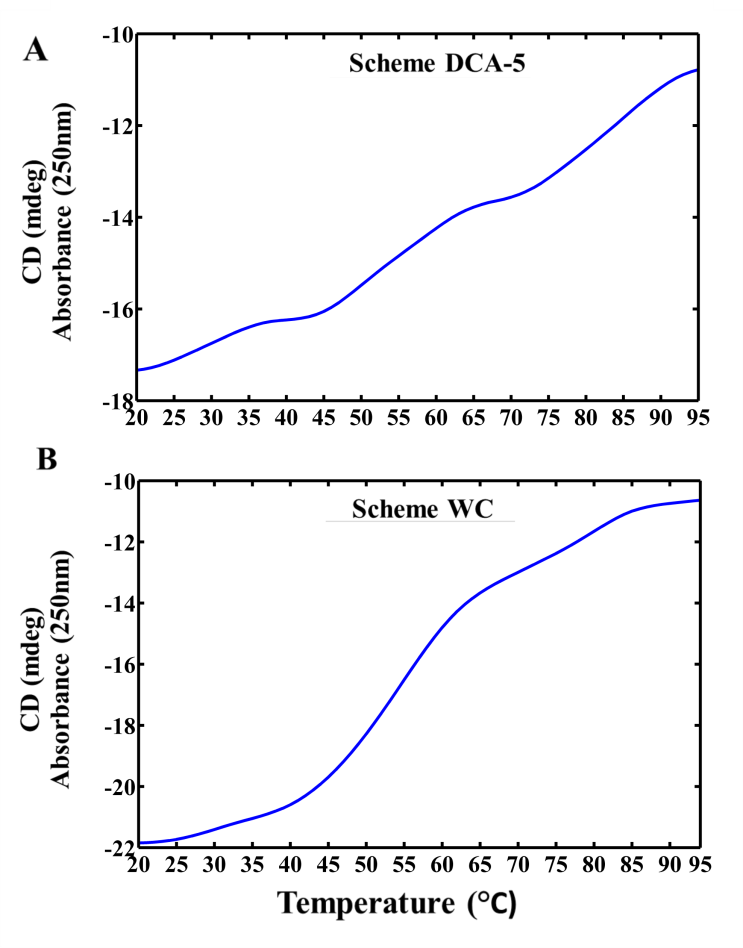** |
| --- |
| **Figure S6. Thermal denaturation profiles of the schemes WC and DCA-5 (Table 1).** (A,B) The thermal denaturation data confirming the formation of a duplex structure by DCA-5 and WC. However, the presence of a biphasic trend is suggestive of multiple conformational preferences. Note that the thermal denaturation is done in triplicate and the average thermal denaturation curve is given here. The MATLAB 7.11.0 software (www.mathworks.com) was used to plot the data. |

| **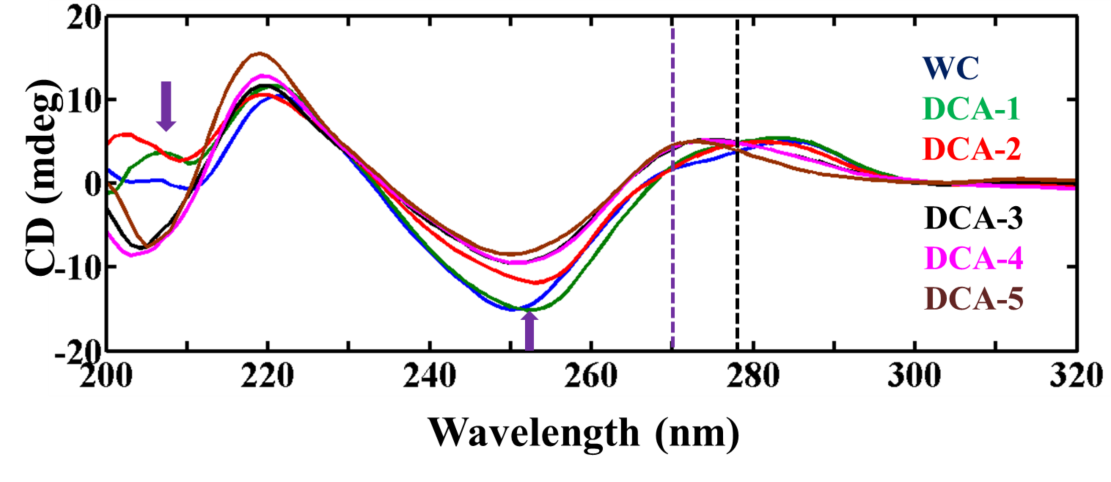** |
| --- |
| **Figure S7. CD spectra (collected at 4M NaCl) of d(CAA) duplexes (Table 1) that have A…A mismatch in the range of 0 to 5.** As the number of A…A mismatch increases, the negative peak around 205nm and 250nm increases and decreases respectively (purple arrows), which is accompanied by a shift in the positive peak from 280nm (black dashed line) to 270nm (purple dashed line). This indicates the increase in the B-Z junction with respect to the increase in the number of A…A mismatch. The MATLAB 7.11.0 software (www.mathworks.com) was used to plot the data. |

| **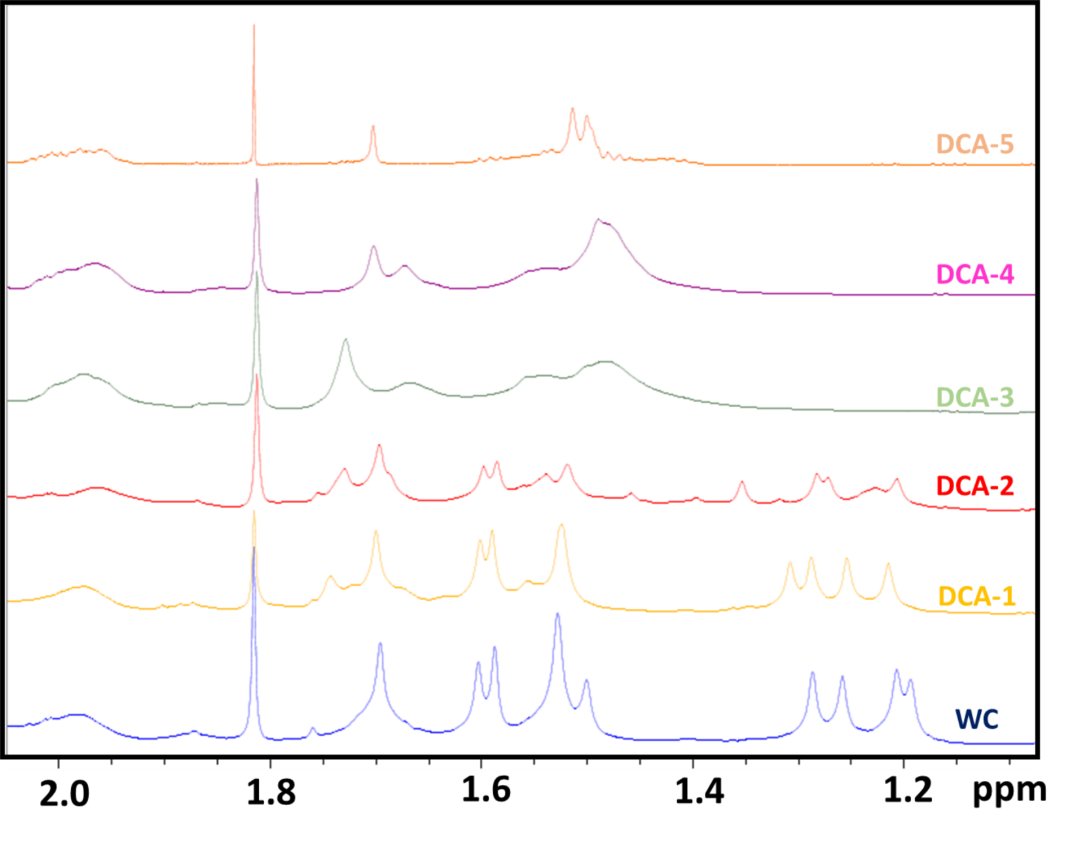** |
| --- |
| **Figure S8. 1D proton NMR spectra corresponding to methyl proton region.** The TopSpin 4.0.2 software (www.bruker.com) was used for processing the NMR data. |

| **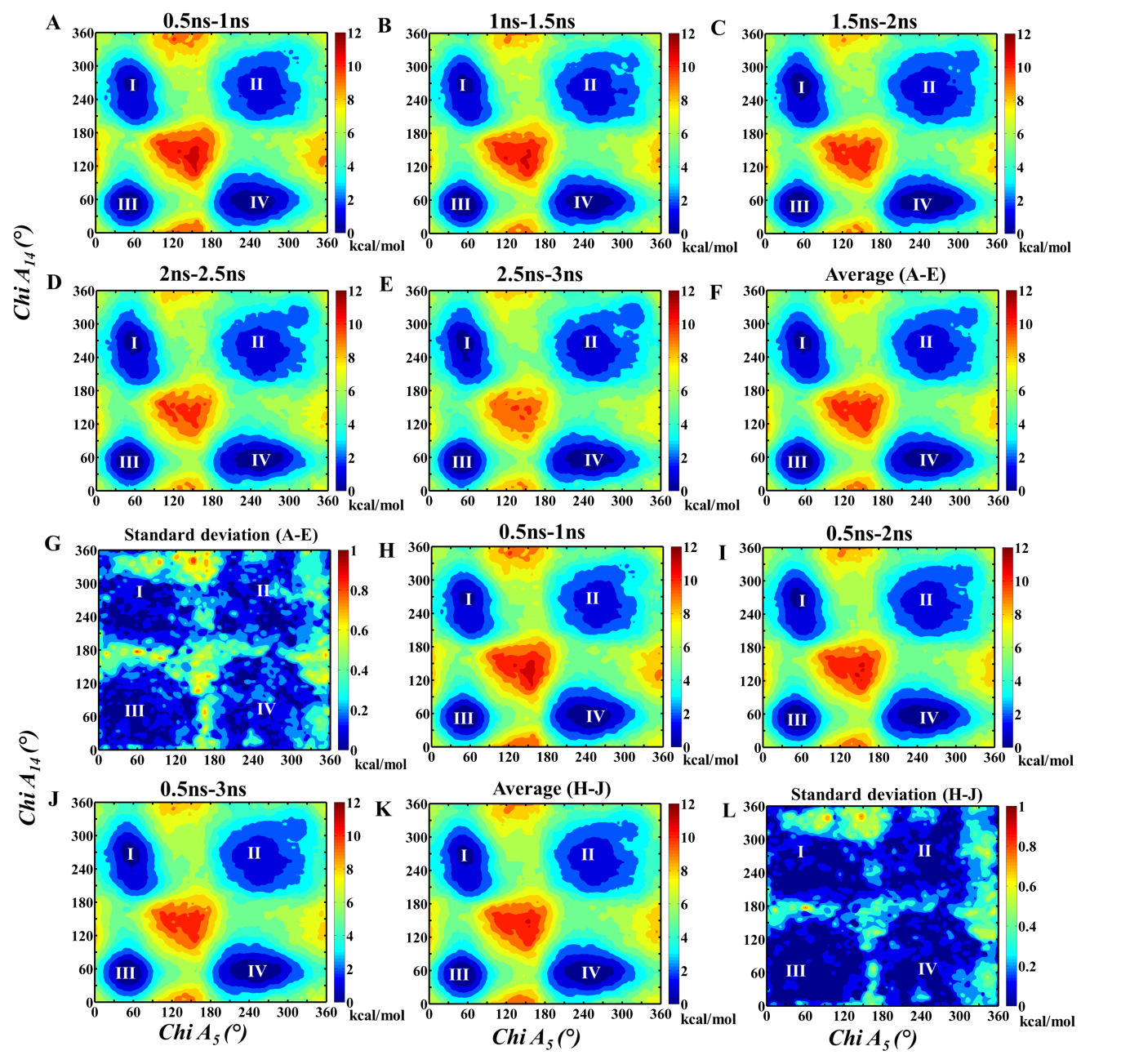** |
| --- |
| **Figure S9. PMF corresponding to different time intervals confirms the presence of 4 minima regions for the scheme DCA-1a. (**A-E) The PMF plotted by considering the time intervals 0.5-1ns, 1-1.5ns, 1.5-2ns, 2-2.5ns and 2.5-3ns. (F) The average of PMFs (A-E) and (G) the corresponding standard deviation. (H-J) The PMF plotted by following a different time interval scheme: 0.5-1ns, 0.5-2ns and 0.5-3ns. (K) The average of PMFs (H-J) and (L) the corresponding standard deviation. The MATLAB 7.11.0 software (www.mathworks.com) was used to plot the data. |

| **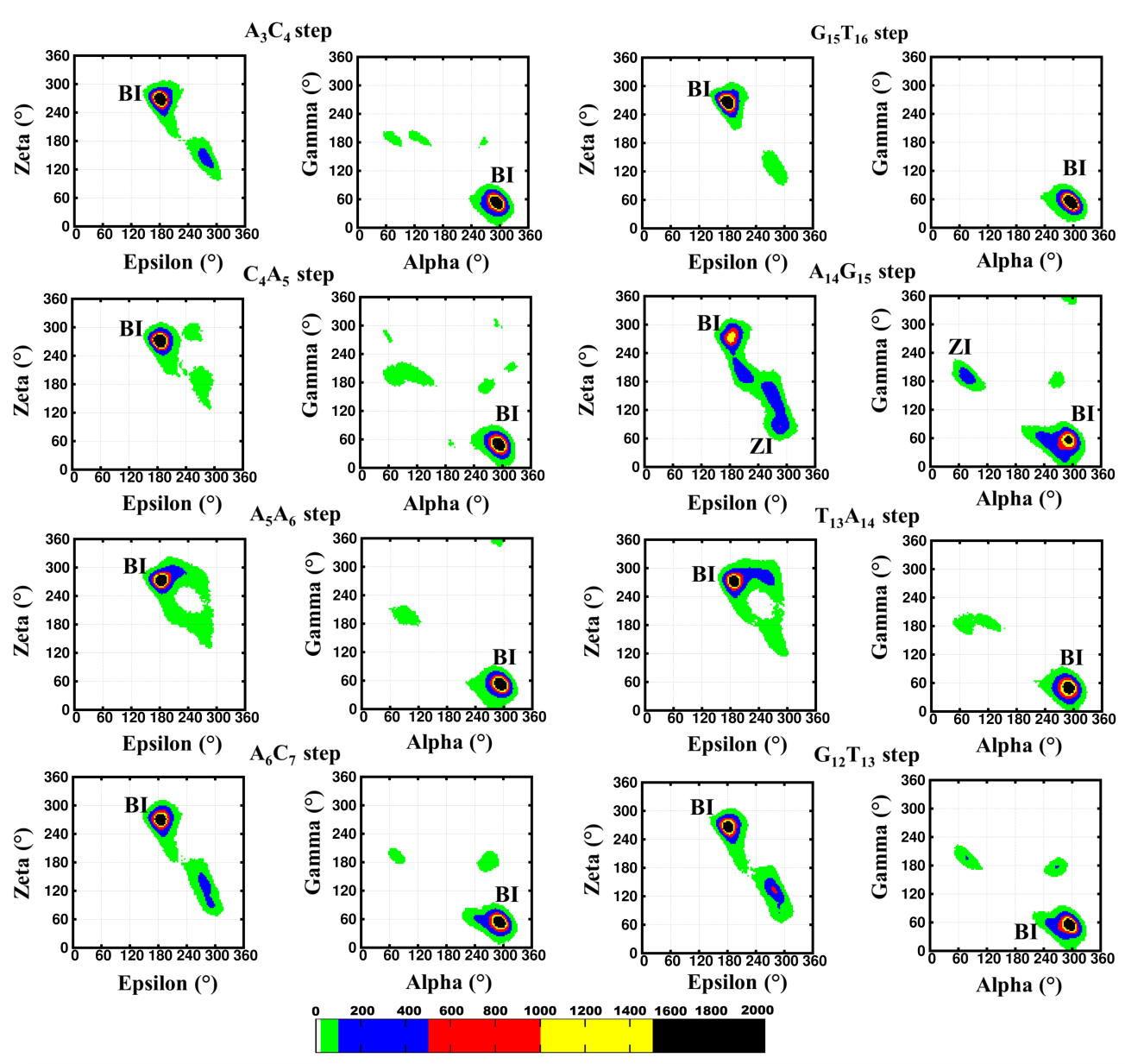** |
| --- |
| **Figure S10. The backbone torsion angles (**ε,ζ,α,γ**) corresponding to the minima region I (**χ_5_(20°-100°)…χ_14_(200°-330°)) **(Figure 6C, Main text) of the umbrella sampling MD (Scheme DCA-1a).** (ε&ζ) (1^st^ and 3^rd^ column) and (α&γ) (2^nd^ and 4^th^ column) 2D contour density plots corresponding to various steps in the vicinity of the mismatch. Note that the BI ((ε,ζ,α,γ)=(t,g^-^,g^-^,g^+^)), BII (g^-^,t,g^-^,g^+^), BIII (g^-^,g^-^,g^-^,g^+^) and ZI (g^-^,g^+^,g^+^,t) conformations are indicated adjacent to the corresponding regions. Other conformational intermediates can also be seen in the plot. The trajectories corresponding to the last 2.5ns of each window are considered for the plotting. The scale corresponding to the isolines is given at the bottom. The GNUPLOT 5.2 software was used to plot the data [2]. |

| **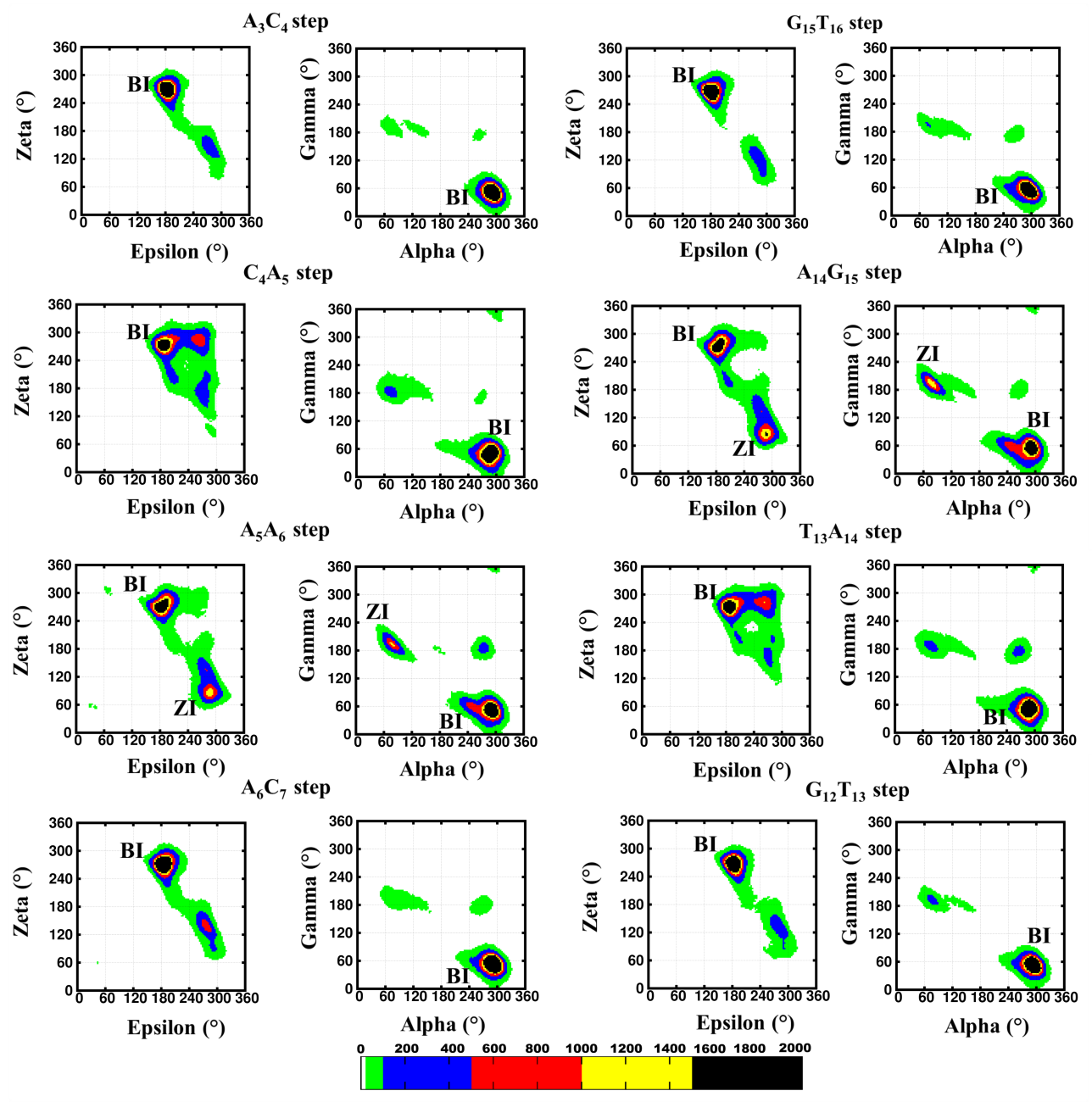** |
| --- |
| **Figure S11. The backbone torsion angles (**ε,ζ,α,γ**) corresponding to the minima region II (**χ_5_(190°-340°)…χ_14_(190°-340°)) **(Figure 6C, Main text) of the umbrella sampling MD (Scheme DCA-1a).** (ε&ζ) (1^st^ and 3^rd^ column) and (α&γ) (2^nd^ and 4^th^ column) 2D contour density plots corresponding to various steps in the vicinity of the mismatch. Note that the BI ((ε,ζ,α,γ)=(t,g^-^,g^-^,g^+^)), BII (g^-^,t,g^-^,g^+^), BIII (g^-^,g^-^,g^-^,g^+^) and ZI (g^-^,g^+^,g^+^,t) conformations are indicated adjacent to the corresponding regions. Other conformational intermediates can also be seen in the plot. The trajectories corresponding to the last 2.5ns of each window are considered for the plotting. The scale corresponding to the isolines is given at the bottom. The scale corresponding to the isolines is given at the bottom. The GNUPLOT 5.2 software was used to plot the data [2]. |

| **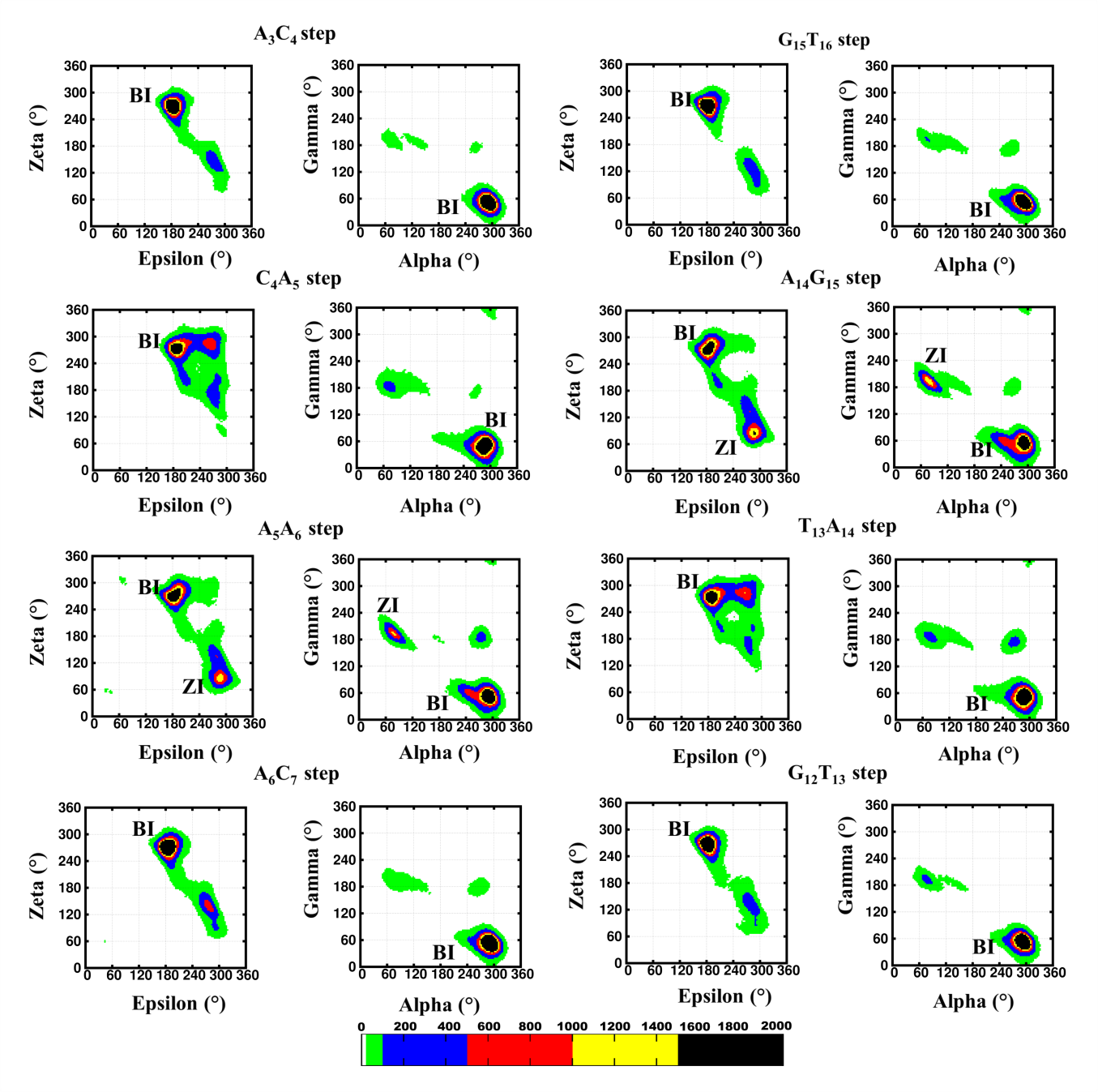** |
| --- |
| **Figure S12. The backbone torsion angles (**ε,ζ,α,γ**) corresponding to the minima region III (**χ_5_(20°-90°)…χ_14_(20°-90°)) **(Figure 6C, Main text) of the umbrella sampling MD (Scheme DCA-1a).** (ε&ζ) (1^st^ and 3^rd^ column) and (α&γ) (2^nd^ and 4^th^ column) 2D contour density plots corresponding to various steps in the vicinity of the mismatch. Note that the BI ((ε,ζ,α,γ)=(t,g^-^,g^-^,g^+^)), BII (g^-^,t,g^-^,g^+^), BIII (g^-^,g^-^,g^-^,g^+^) and ZI (g^-^,g^+^,g^+^,t) conformations are indicated adjacent to the corresponding regions. Other conformational intermediates can also be seen in the plot. The trajectories corresponding to the last 2.5ns of each window are considered for the plotting. The scale corresponding to the isolines is given at the bottom. The scale corresponding to the isolines is given at the bottom. The GNUPLOT 5.2 software was used to plot the data [2]. |

| **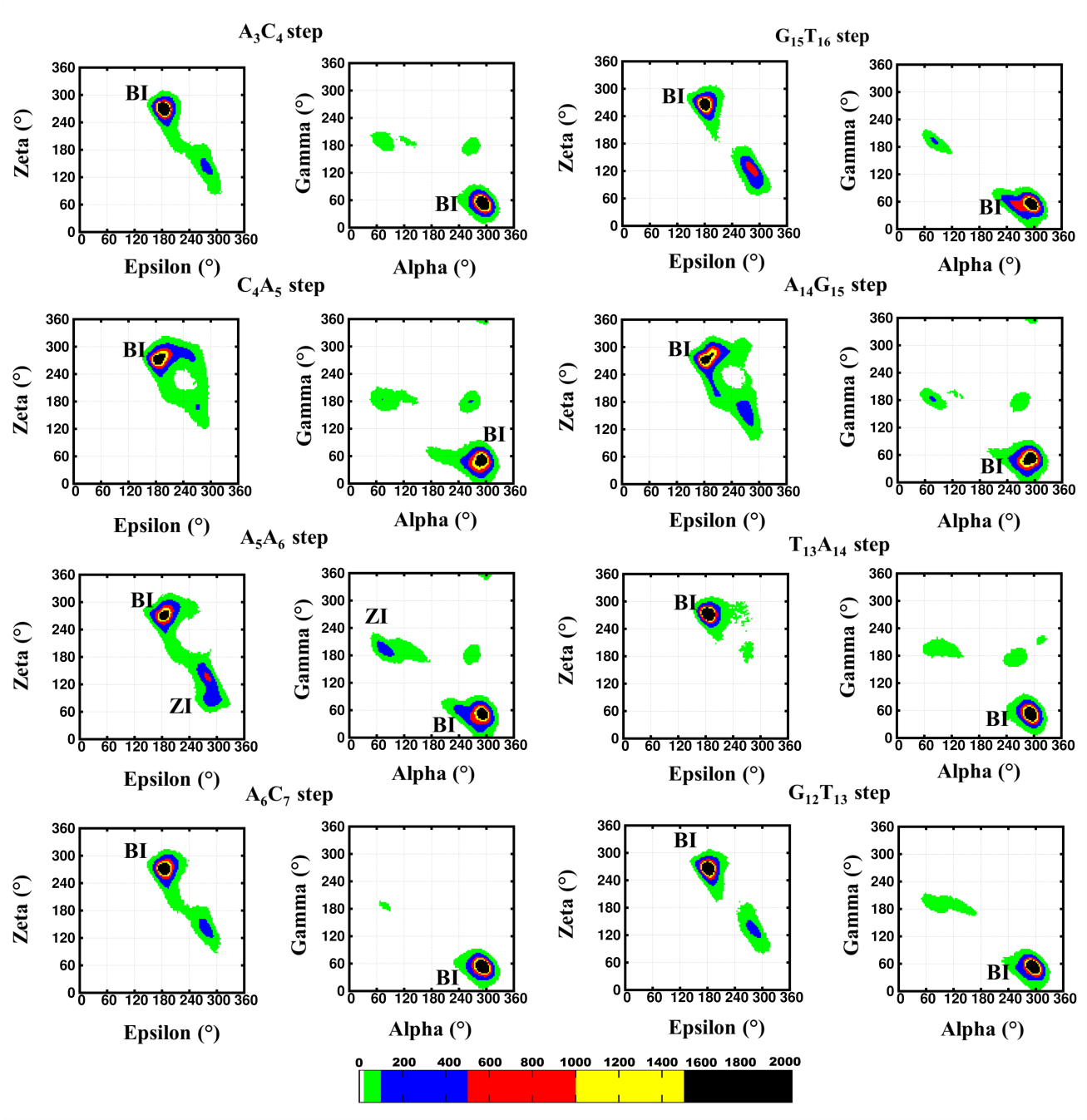** |
| --- |
| **Figure S13. The backbone torsion angles (**ε,ζ,α,γ**) corresponding to the minima region IV** (χ_5_(180°-330°)…χ_14_(20°-100°)) **(Figure 6C, Main text) of the umbrella sampling MD (Scheme DCA-1a).** (ε&ζ) (1^st^ and 3^rd^ column) and (α&γ) (2^nd^ and 4^th^ column) 2D contour density plots corresponding to various steps in the vicinity of the mismatch. Note that the BI ((ε,ζ,α,γ)=(t,g^-^,g^-^,g^+^)), BII (g^-^,t,g^-^,g^+^), BIII (g^-^,g^-^,g^-^,g^+^) and ZI (g^-^,g^+^,g^+^,t) conformations are indicated adjacent to the corresponding regions. Other conformational intermediates can also be seen in the plot. The trajectories corresponding to the last 2.5ns of each window are considered for the plotting. The scale corresponding to the isolines is given at the bottom. The scale corresponding to the isolines is given at the bottom. The GNUPLOT 5.2 software was used to plot the data [2]. |

| **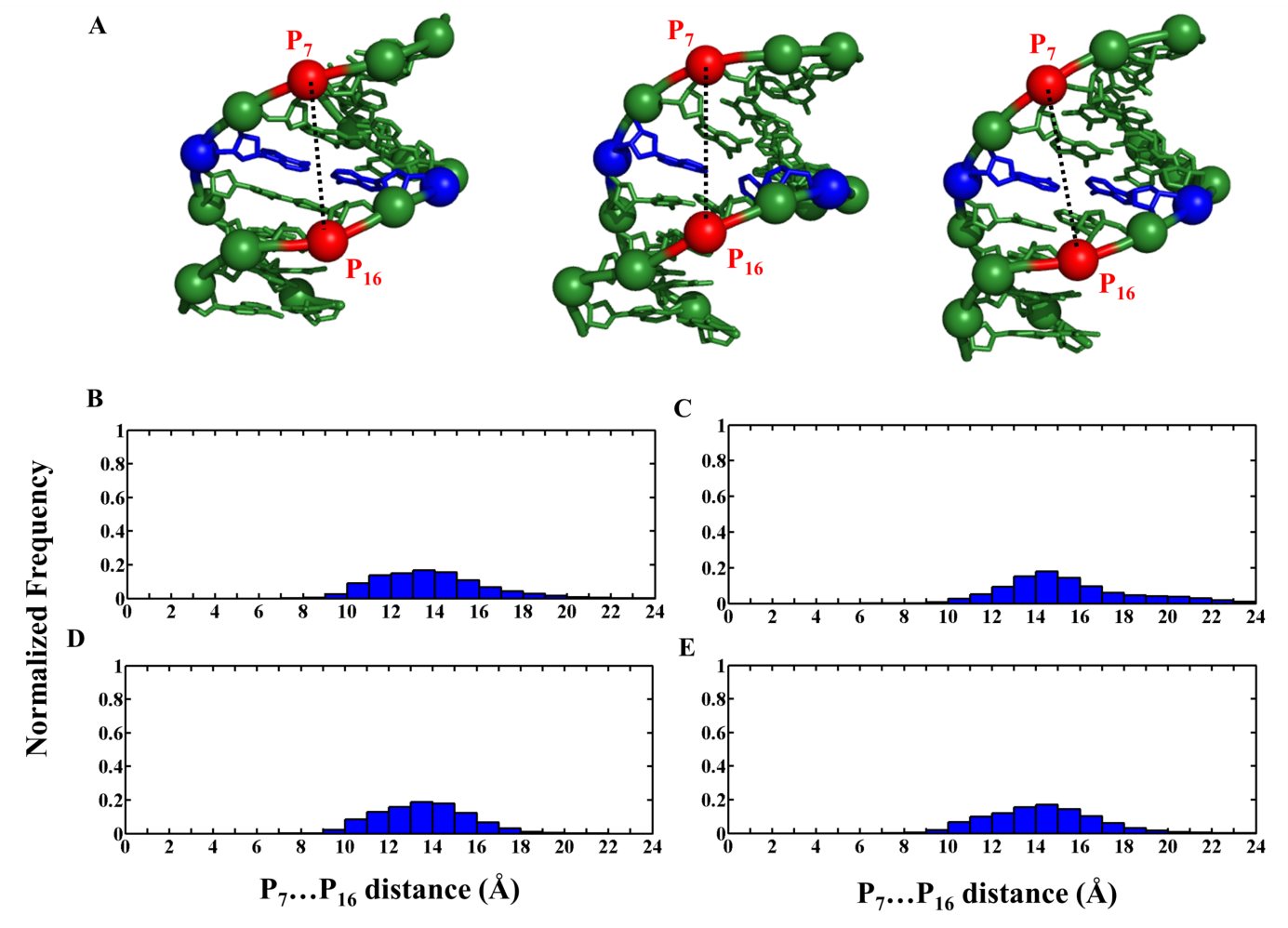** |
| --- |
| **Figure S14.** **Widening of the minor groove at the mismatch site during the umbrella sampling simulation**. **(A)** Cartoon diagram illustrating the widening of the minor groove. The dotted black line represents the P_7_...P_16_ (red colored spheres) distance. This figure was generated by using Pymol 1.3 (www.pymol.com). Note that the A_5_...A_14_ are colored blue. P_7_…P_16_ distance histogram corresponding to the minima regions I **(B)**, II **(C)**, III **(D)** and IV **(E)**. The figures B-E were plotted by using MATLAB 7.11.0 software (www.mathworks.com). |

| **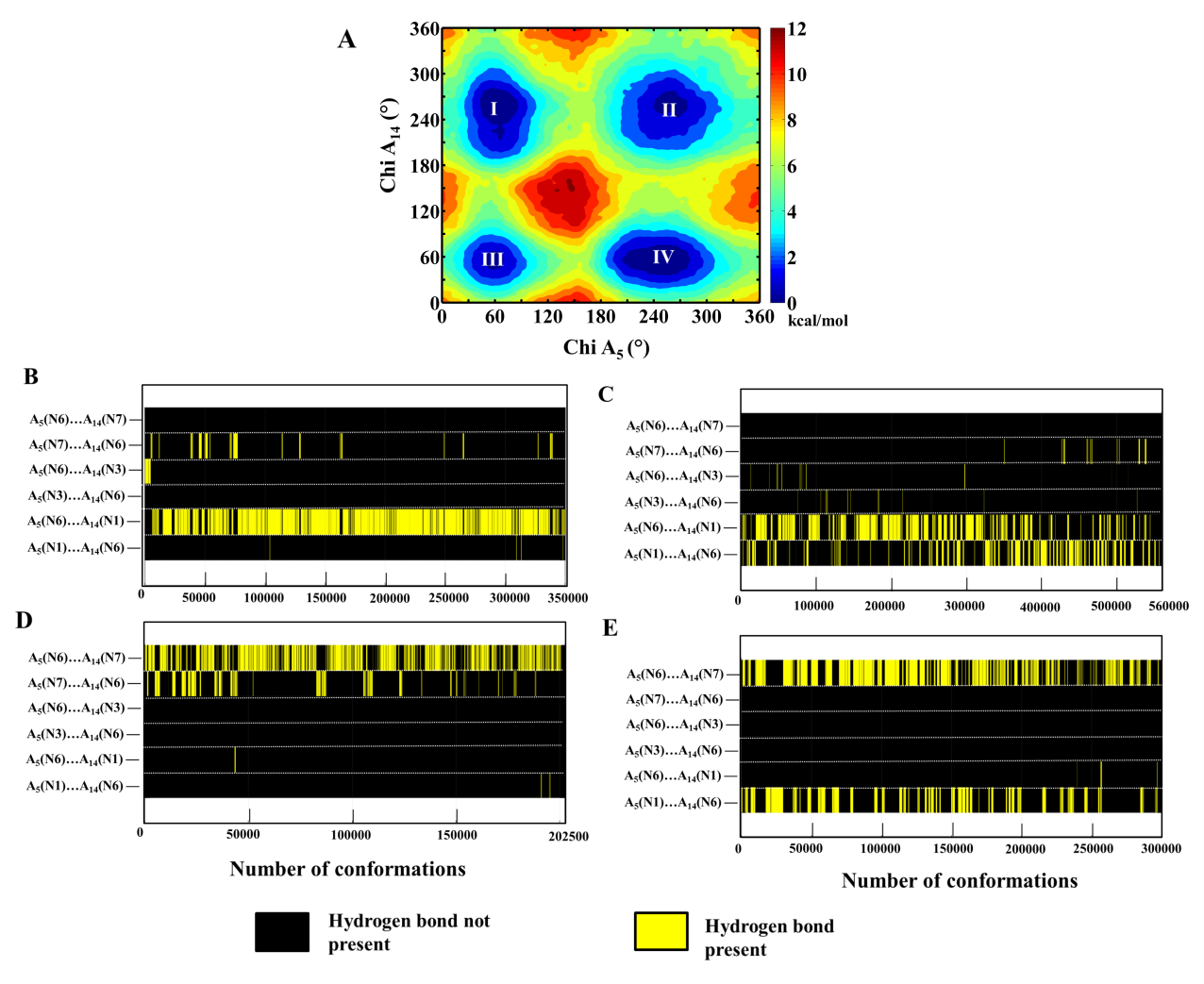** |
| --- |
| **Figure S15. Umbrella sampling simulation corresponding to the scheme DAC-1a (Table 1) that has a single A_5_…A_14_ mismatch in the midst of 5’A…T and 3’C…G.** (A) The 2D free energy map constructed by considering χ_5_ and χ_14_ as reaction coordinates. The labels I-IV indicate the favorable minima regions. The region I corresponds to χ_5_(20°-110°)…χ_14_(190°-320°) and the region II corresponds to χ_5_(190°-340°)…χ_14_(190°-320°). Similarly, regions III and IV represent the conformational spaces corresponding to χ_5_(20°-100°)…χ_14_(20°-100°) and χ_5_(190°-330°)…χ_14_(20°-90°) respectively. The MATLAB 7.11.0 software (www.mathworks.com) was used to plot the data. (B-E) Hydrogen bond life time profiles corresponding to the energetically favored regions of the free energy map (A): (B) +*syn…anti* (χ_5_ and χ_14_ each has 10x14=140 windows in this region, wherein, each window has 2500 frames), (C) *anti…anti,* (D) *+syn…+syn* and (E) *anti…+syn* regions. Note that the windows (each window has 2500 frames) corresponding to each region are arranged adjacent to each other and are represented in terms of total number of frames along the X-axis. The GNUPLOT 5.2 software was used to plot the data [2]. |

| **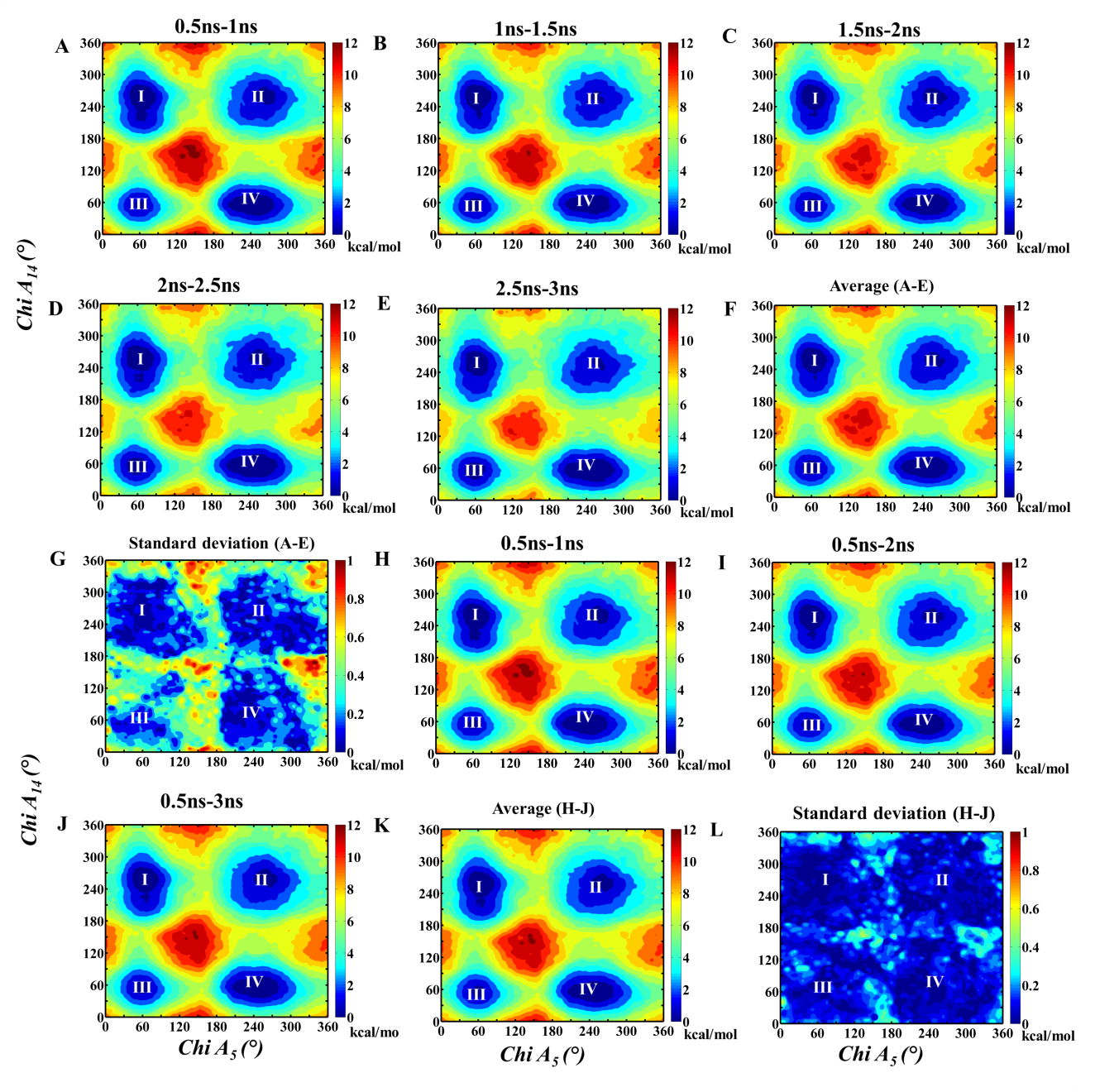** |
| --- |
| **Figure S16. PMF corresponding to different time intervals confirms the presence of 4 minima regions for the scheme DAC-1a. (**A-E) The PMF plotted by considering the time intervals 0.5-1ns, 1-1.5ns, 1.5-2ns, 2-2.5ns and 2.5-3ns. (F) The average of PMFs (A-E) and (G) the corresponding standard deviation. (H-J) The PMF plotted by following a different time interval scheme: 0.5-1ns, 0.5-2ns and 0.5-3ns. (K) The average of PMFs (H-J) and (L) the corresponding standard deviation. The MATLAB 7.11.0 software (www.mathworks.com) was used to plot the data. |

| **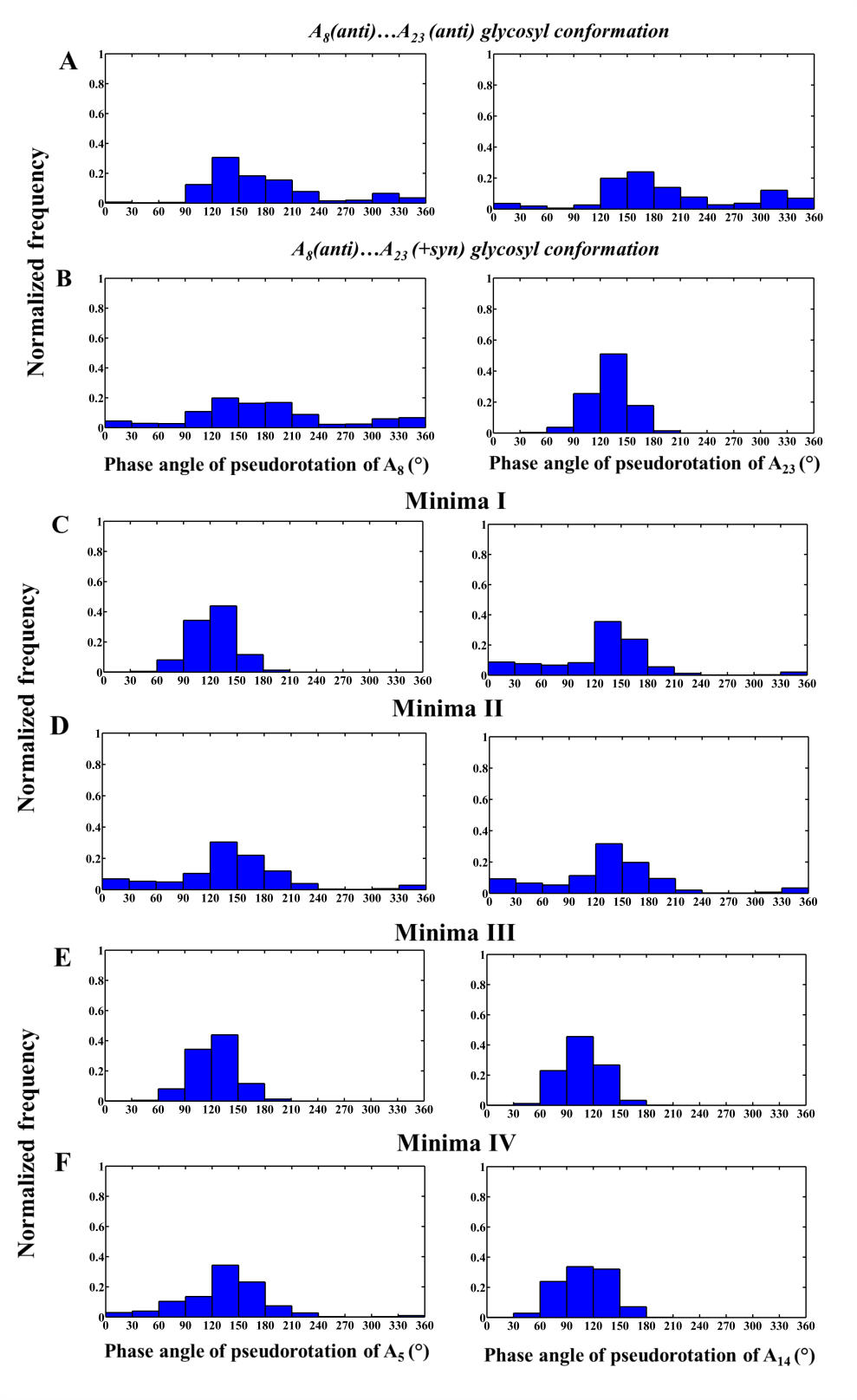** |
| --- |
| **Figure S17. Sugar puckering analysis corresponding to the schemes DCA-1 (MD simulation) and DCA-1a (umbrella sampling MD) (Table 1).**  Phase angle of pseudo-rotation versus normalized frequency histogram corresponding to the MD simulations with (A) *anti…anti* & (B) *anti…+syn* starting *glycosyl* conformations (scheme DCA-1). (C-F) Phase angle of pseudo-rotation versus normalized frequency histogram corresponding to the umbrella sampling MD (scheme DCA-1a). The MATLAB 7.11.0 software (www.mathworks.com) was used to plot the data. |

| *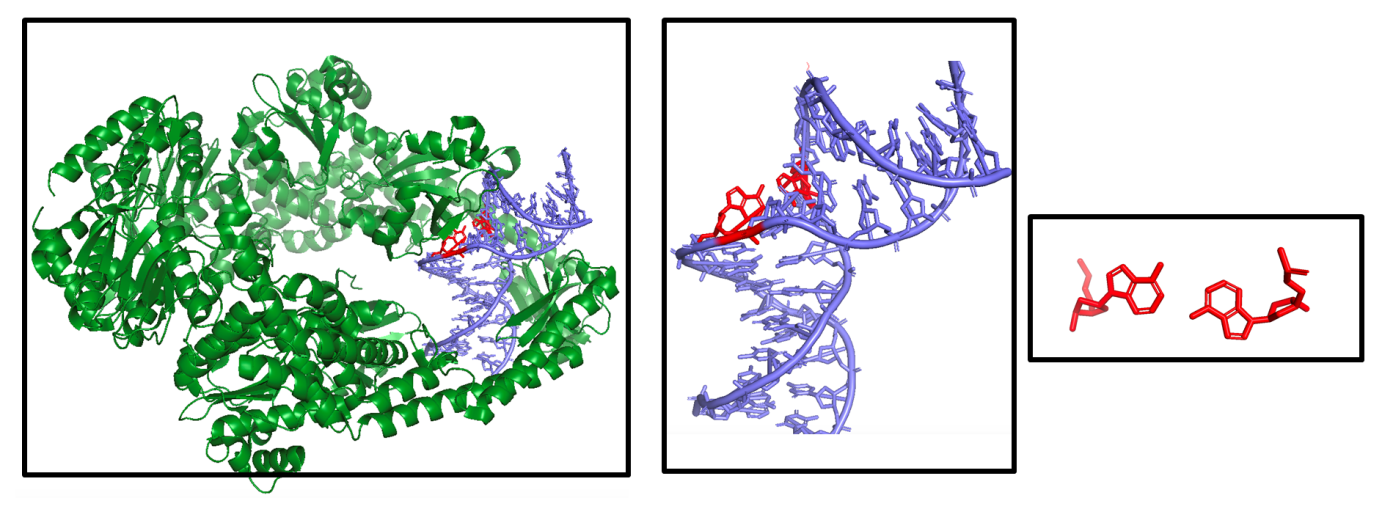* |
| --- |
| **Figure S18**. **Crystal structure of *E. coli* mismatch repair protein MutS (colored green) complex (Left) with a DNA duplex (colored blue) having an A…A mismatch (colored red) (PDB ID:1OH6).** The duplex (middle) and the mismatch (right) are zoomed and shown separately. Note the movement of N6 of one of the adenines (Right) towards the minor groove. |

**References**

1. Humphrey, W., A. Dalke, and K. Schulten, *VMD: visual molecular dynamics.* J Mol Graph, 1996. **14**(1): p. 33-8, 27-8.

2. Williams, T., et al., *gnuplot 5.2*. 2017.
